# Design of 4-Substituted Sulfonamidobenzoic Acid Derivatives Targeting Coxsackievirus B3

**DOI:** 10.3390/life12111832

**Published:** 2022-11-09

**Authors:** Anton A. Shetnev, Alexandrina S. Volobueva, Valeria A. Panova, Vladimir V. Zarubaev, Sergey V. Baykov

**Affiliations:** 1 Pharmaceutical Technology Transfer Center, Yaroslavl State Pedagogical University Named after K.D. Ushinsky, 108 Respublikanskaya St., 150000 Yaroslavl, Russia; 2 Saint Petersburg Pasteur Institute, 14 Mira Street, 197101 Saint Petersburg, Russia; 3 Institute of Chemistry, Saint Petersburg State University, 7/9 Universitetskaya Nab., 199034 Saint Petersburg, Russia

**Keywords:** enteroviruses, capsid binders, screening, imide, aminobenzoic acid, heterocycles

## Abstract

A series of novel 4-substituted sulfonamidobenzoic acid derivatives was synthesized as the structural evolution of 4-(4-(1,3-dioxoisoindolin-2-yl)phenylsulfonamido)benzoic acid, which is the known inhibitor of the enterovirus life cycle. Antiviral properties of prepared compounds were evaluated *in vitro* using phenotypic screening and viral yield reduction assay. Their capsid binding properties were verified in thermostability assay. We identified two new hit-compounds (**4** and **7a**) with high activity against the coxsackievirus B3 (Nancy, CVB3) strain with potencies (IC_50_ values of 4.29 and 4.22 μM, respectively) which are slightly superior to the reference compound **2a** (IC_50_ 5.54 μM). Both hits changed the heat inactivation of CVB3 *in vitro* to higher temperatures, suggesting that they are capsid binders, as **2a** is. The results obtained can serve as a basis for further development of the lead compounds for novel drug design to combat enterovirus infection.

## 1. Introduction

Viruses of the genus Enterovirus belonging to the *Picornaviridae* family are small, non-enveloped viruses with genomes represented by a single-stranded (+) RNA molecule. According to the modern classification of the International Committee on the Taxonomy of Viruses (ICTV), within the genus enterovirus, four types of enteroviruses (A-D) and three types of rhinoviruses (A–C) are distinguished [1]. Enteroviruses are etiological agents of acute and chronic human diseases, from asymptomatic or mild infection to life-threatening myocarditis, acute flaccid myelitis, cardiomyopathy, poliomyelitis, encephalitis, meningitis, bronchiolitis and pneumonia [2]. When entering the human body, they multiple mainly in the gastrointestinal tract and/or upper respiratory tract, but can penetrate into other tissues and target organs from primary infection sites viruses, causing severe complications. Rhinoviruses are the main cause of the common cold, a mild upper respiratory illness, but they can also provide lower respiratory illnesses such as bronchitis, sinusitis, and pneumonia, as well as worsening asthma, cystic fibrosis, and chronic obstructive pulmonary disease [3].

Enteroviruses are characterized by genetic plasticity due to recombination, high resistance to environmental conditions and the ability to cause outbreaks (in particular in organized groups of children) and epidemics. During the last decade, an outbreak of enterovirus D68 in 2014–2016 in the USA led to severe respiratory diseases in children, which in many cases were associated with acute flaccid myelitis [4]. Enterovirus 71 (EV71) infections are of high importance in the Asia-Pacific region [5].

The development of vaccines to prevent human enterovirus and rhinovirus infection is complicated by the multiplicity of existing virus serotypes. To date, there are vaccines for the prevention of poliomyelitis (live and inactivated) and three inactivated EV-A71 vaccines. The later ones are approved by the National Medical Products Administration of China (NMPA) and are commercially available in China [6]. The enormous efforts of the Global Polio Eradication Initiative have been very successful. However, it is now generally accepted that antivirals will be required for successful polio eradication, in particular to control outbreaks of circulating vaccine-derived polioviruses and to stop the excretion of chronically shedding viruses [7].

Despite significant advances in the field of enteroviruses and rhinoviruses biology, there are currently no approved drugs for the treatment and prevention of diseases caused by these viruses [8]. Among the previously studied enterovirus and rhinovirus inhibitor molecules with a known mechanism of action and documented activity in studies on animal models, the following groups can be distinguished: inhibitors that bind to the viral capsid and prevent its penetration into the cell—capsid binders (pleconaril, pirodavir, vapendavir, pokapavir, disoxaril), viral protease inhibitors (rupintrivir and AG7404), virus polymerase inhibitors (ribavirin, gemcitabine, amiloride), and viral ATPase inhibitors (dibucaine, fluoxetine) [9,10]. Despite being active in preclinical studies, none have been approved due to lack of efficacy, poor pharmacokinetic profile and/or adverse effects in clinical studies [11]. It should be noted that the potential use of drugs originally used for other medical conditions (e.g., fluoxetine, an antidepressant, amiloride, a diuretic) has the potential to cause unpredictable emergence of resistant strains in cases where their intended use coincides with asymptomatic EVI in a patient. Thus, the development of effective direct antivirals targeting enterovirus and rhinovirus lifecycle is highly desirable due to unmet clinical needs.

Capsid binders represent the most investigated group of inhibitors targeting viruses of the Enterovirus genus. Their general mechanism of action is well understood: they bind to a virus capsid, causing conformational changes that disturb the binding of the virus to its receptor on the cell surface [12]. These compounds have a pronounced antiviral activity, but, unfortunately, it has been shown that their use in clinical studies is associated with the emergence of resistant strains [13]. Nevertheless, given the importance of the initial step in the virus life-cycle, the search for targets on the surface of the virus for the design of inhibitors is still promising.

**Figure 1:**
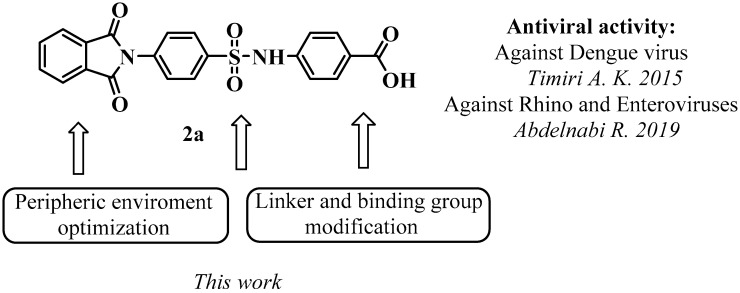
General purpose for medicinal-chemical modification of lead-compound **2a**. The antiviral activity was investigated in Refs. [14,15].

For a long time, it was believed that the hydrophobic pocket at the bottom of the canyon formed around each of the 12 vertices of the icosahedral viral capsid is the only target for capsid binder design. However, in 2019 a new promising target on the surface of the capsid was identified through the collaboration of the Neyts, Butcher, and Sinha research groups [14]. This pocket is located between the VP1-VP3 protomers in a conserved region of the surface of the enterovirus capsid. It was shown that the following amino acid residues line the pocket: VP1 (73, 75–78, 155–157, 159–160, 219, and 234) and VP3 (233–236). The residues support quaternary structural changes in the capsid during virus entry when the virion progresses to an expanded altered particle (A-particle). This pocket was found to be suitable as a target for blocking the life cycle of enteroviruses and rhinoviruses. The identification of the hit compound (4-substituted sulfonamidobenzoic acid, Figure 1) carried out in the same study and the published electron microscopy data on the structure of a new pocket on the surface of the enterovirus capsid created the prerequisites for further successful chemical research in the field of capsid binders.

In this study, we explored the possibilities for medicinal-chemical modification of the lead-compound **2a** [14] to identify new highly active molecules that exhibit the properties of new capsid-binding agents for the prevention and treatment of infections caused by enteroviruses. The results obtained can be used to develop new capsid-binding agents for the prevention and treatment of diseases caused by enteroviruses.

## 2. Materials and Methods

### 2.1. General

The preparation and characterization of starting sulfonyl chloride **1** as well as amine **5** were described previously [15,16,17]. Other reactants and solvents were obtained from commercial sources and used without purification. All reactions were performed in an open flask without any protection from CO_2_ and H_2_O. Reactions were monitored using analytical thin layer chromatography (TLC) Macherey-Nagel, TLC plates Polygram^®^ Sil G/UV254. Visualization of the developed chromatograms was performed by fluorescence quenching at 254 nm. ^1^H and ^13^C NMR spectra were measured on Bruker AVANCE DPX 400 (400 MHz for ^1^H and 101 MHz for ^13^C, respectively). All chemical shifts (δ) are given in parts per million (ppm) with reference to solvent residues in DMSO-*d*_6_ (2.50 for proton and 39.52 for carbon), and coupling constants (*J)* are reported in hertz (Hz). Multiplicities are abbreviated as follows: s = singlet, d = doublet, t = triplet, q = quartet, m = multiplet, br = broad. Melting points were determined using an Electrothermal IA 9300 series Digital Melting Point Apparatus. Mass spectra were recorded on microTOF spectrometers (ESI ionization).

### 2.2. Synthesis and Characterization of Compounds ***2a**–**i***

Pyridine (1.05 mmol) and 4-(1,3-dioxoisoindolin-2-yl)benzenesulfonyl chloride **1** (1 mmol) were added to a solution of the corresponding amine (1 mmol) in acetonitrile (2 mL). The mixture was stirred at room temperature for 6–12 h (TLC monitoring). Acetonitrile was removed in vacuo and the residue was treated with water (10 mL), and the precipitate was filtered off and dried. Compounds **2b**, **2d**, and **2i** were isolated in acceptable purity, whereas other compounds (**2a**, **2c**, **2e**–**h**) were recrystallized from ethanol.

***4-(4-(1,3-Dioxoisoindolin-2-yl)phenylsulfonamido)benzoic acid******2a** [13]*: Yield 63%, white powder, mp 263–265 °C. ^1^H NMR (400 MHz, DMSO-*d*_6_) δ: 12.75 (br.s, 1 H), 10.95 (s, 1 H), 8.02–7.97 (m, 4 H), 7.93–7.89 (m, 2 H), 7.83 (d, *J* = 8.3 Hz, 2 H), 7.70 (d, *J* = 8.4 Hz, 2 H), 7.25 (d, *J* = 8.6 Hz, 2 H). ^13^C NMR (101 MHz, DMSO-*d*_6_) δ: 167.36 (C=O), 167.10 (2 C=O), 142.46, 138.88, 136.71, 135.58 (2 C), 132.06 (2 C), 131.50 (2 C), 128.25 (2 C), 128.07 (2 C), 126.45, 124.28 (2 C), 118.82 (2 C).

***Ethyl 4-(4-(1,3-dioxoisoindolin-2-yl)phenylsulfonamido)benzoate******2b**:* Yield 80%, light pink powder, mp 255–257 °C. ^1^H NMR (400 MHz, DMSO-*d*_6_) δ: 10.99 (s, 1 H), 8.02–7.96 (m, 4 H), 7.95–7.88 (m, 2 H), 7.85 (d, *J* = 8.8 Hz, 2 H), 7.69 (d, *J* = 8.6 Hz, 2 H), 7.28 (d, *J* = 8.8 Hz, 2 H), 4.24 (q, *J* = 7.1 Hz, 2 H), 1.26 (t, *J* = 7.1 Hz, 3 H). ^13^C NMR (101 MHz, DMSO-*d*_6_) δ: 167.10 (2 C=O), 165.78 (C=O), 142.77, 138.78, 136.74, 135.58 (2 C), 132.07 (2 C), 131.34 (2 C), 128.26 (2 C), 128.09 (2 C), 125.54, 124.28 (2 C), 118.87 (2 C), 61.17 (CH_2_), 14.83 (CH_3_). HRMS (ESI), Calc. for C_23_H_19_N_2_O_6_S^+^: [M+H]^+^ 451.0958; found *m*/*z* 451.0966.

***4-(4-(1,3-Dioxoisoindolin-2-yl)phenylsulfonamido)benzamide 2c****:* Yield 90%, white powder, mp 240–242 °C. ^1^H NMR (400 MHz, DMSO-*d*_6_) δ: 10.78 (s, 1 H), 8.01–7.94 (m, 4 H), 7.91 (dd, *J* = 5.5, 3.1 Hz, 2 H), 7.82 (s, 1 H), 7.75 (d, *J* = 8.4 Hz, 2 H), 7.69 (d, *J* = 8.3 Hz, 2 H), 7.24 (s, 1 H), 7.19 (d, *J* = 8.3 Hz, 2 H). ^13^C NMR (101 MHz, DMSO-*d*_6_) δ: 167.87 (CONH_2_), 167.12 (2 C=O), 140.93, 138.95, 136.62, 135.59 (2 C), 132.08 (2 C), 130.28, 129.52 (2 C), 128.24 (2 C), 128.06 (2 C), 124.29 (2 C), 118.91 (2 C). HRMS (ESI), Calc. for C_21_H_16_N_3_O_5_S^+^: [M+H]^+^ 422.0805; found *m/z* 422.0816.

***N-(4-Cyanophenyl)-4-(1,3-dioxoisoindolin-2-yl)benzenesulfonamide 2d****:* Yield 90%, white powder, mp 224–226 °C. ^1^H NMR (400 MHz, DMSO-*d*_6_) δ: 11.20 (s, 1 H), 8.01 (d, *J* = 8.6 Hz, 2 H), 8.01–7.93 (m, 4 H), 7.74–7.70 (m, 4 H), 7.31 (d, *J* = 8.7 Hz, 2 H). ^13^C NMR (101 MHz, DMSO-*d*_6_) δ: 167.10 (2 C=O), 142.75, 138.63, 136.90, 135.60 (2 C), 134.47 (2 C), 132.09 (2 C), 128.37 (2 C), 128.09 (2 C), 124.30 (2 C), 119.34, 119.19 (2 C), 106.26 (C≡N). HRMS (ESI), Calc. for C_21_H_14_N_3_O_4_S^+^: [M+H]^+^ 404.0700; found *m/z* 404.0707.

***4-(1,3-Dioxoisoindolin-2-yl)-N-(4-sulfamoylphenyl)benzenesulfonamide 2e****:* Yield 68%, white powder, mp 261–263 °C. ^1^H NMR (400 MHz, DMSO-*d*_6_) δ: 10.95 (s, 1 H), 8.03–7.95 (m, 4 H), 7.91 (dd, *J* = 5.5, 3.1 Hz, 2 H), 7.73–7.69 (m, 4 H), 7.32 (d, *J* = 8.8 Hz, 2 H), 7.20 (s, 2 H). ^13^C NMR (101 MHz, DMSO-*d*_6_) δ: 167.14 (2 C=O), 141.38, 139.65, 138.75, 136.76, 135.60 (2 C), 132.07(2 C), 128.32 (2 C), 128.13 (2 C), 127.97 (2 C), 124.29 (2 C), 119.10 (2 C). HRMS (ESI), Calc. for C_20_H_16_N_3_O_6_S_2_^+^: [M+H]^+^ 458.0475; found *m/z* 458.0489. 

*(**N-(4-(4-(1,3-Dioxoisoindolin-2-yl)phenylsulfonamido)phenyl)acetamide 2f**:* Yield 81%, white powder, mp 220–222 °C. ^1^H NMR (400 MHz, DMSO-*d*_6_) δ: 10.18 (s, 1 H), 9.86 (s, 1 H), 8.00–7.93 (m, 4 H), 7.86 (d, *J* = 8.7 Hz, 2 H), 7.65 (d, *J* = 8.7 Hz, 2 H), 7.44 (d, *J* = 8.9 Hz, 2 H), 7.04 (d, *J* = 8.9 Hz, 2 H), 1.98 (s, 3H). ^13^C NMR (101 MHz, DMSO-*d*_6_) δ: 168.73 (C=O), 167.15 (2 C=O), 139.10, 136.86, 136.30, 135.57 (2 C), 132.88, 132.09 (2 C), 128.03 (4 C), 124.26 (2 C), 122.17 (2 C), 120.41 (2 C), 24.53 (CH_3_). HRMS (ESI), Calc. for C_22_H_18_N_3_O_5_S^+^: [M+H]^+^ 436.0962; found *m/z* 436.0969. 

***1-((4-(1,3-Dioxoisoindolin-2-yl)phenyl)sulfonyl)piperidine-4-carboxylic acid 2g****:* Yield 52%, white powder, mp 207–210 °C. ^1^H NMR (400 MHz, DMSO-*d*_6_) δ: 12.27 (br.s, 1 H), 8.02–7.98 (m, 2 H), 7.95–7.92 (m, 2 H), 7.91 (d, *J* = 8.3 Hz, 2 H), 7.79 (d, *J* = 8.3 Hz, 2 H), 3.53 (d, *J* = 11.7 Hz, 2 H), 2.53–2.47 (m, 2 H), 2.34 (t, *J* = 11.1 Hz, 1 H), 1.91 (dd, *J* = 13.2, 3.8 Hz, 2 H), 1.58 (td, *J* = 14.4, 13.6, 6.6 Hz, 2 H). ^13^C NMR (101 MHz, DMSO-*d*_6_) δ: 175.86 (C=O), 167.20 (2 C=O), 136.67, 135.60 (2 C), 135.24, 132.13 (2 C), 128.80 (2 C), 128.18 (2 C), 124.30 (2 C), 45.90 (2 CH_2_), 39.36 (CH), 27.90 (2 CH_2_). HRMS (ESI), Calc. for C_20_H_19_N_2_O_6_S^+^: [M+H]^+^ 415.0958; found *m/z* 415.0972. 

***1-((4-(1,3-Dioxoisoindolin-2-yl)phenyl)sulfonyl)piperidine-4-carboxamide 2h****:* Yield 54%, white powder, mp 217–220 °C. ^1^H NMR (400 MHz, DMSO-*d*_6_) δ: 8.03–7.98 (m, 2 H), 7.96–7.93 (m, 2 H), 7.91 (d, *J* = 8.3 Hz, 2 H), 7.77 (d, *J* = 8.3 Hz, 2 H), 7.20 (s, 1 H), 6.82 (s, 1 H), 3.62 (d, *J* = 11.6 Hz, 2 H), 2.36 (t, *J* = 11.7 Hz, 2 H), 2.10 (dd, *J* = 13.5, 8.8 Hz, 1 H), 1.80 (d, *J* = 11.9 Hz, 2 H), 1.57 (qd, *J* = 12.8, 4.4 Hz, 2 H). ^13^C NMR (101 MHz, DMSO-*d*_6_) δ: 175.48 (C=O), 166.52 (2 C=O), 135.97, 134.91 (2 C), 134.44, 131.45 (2 C), 128.13 (2 C), 127.47 (2 C), 123.62 (2 C), 45.47 (2 C), 40.08 (CH), 27.72 (2 C). HRMS (ESI), Calc. for C_20_H_19_N_3_O_5_S^+^: [M+H]^+^ 414.1118; found *m/z* 414.1122. 

***4-(4-(1,3-Dioxoisoindolin-2-yl)phenylsulfonamido)butanoic acid******2i**:* Yield 42%, white powder, mp 160–162 °C. ^1^H NMR (400 MHz, DMSO-*d*_6_) δ: 12.09 (s, 1 H), 8.02–7.97 (m, 2 H), 7.96–7.91 (m, 4 H), 7.78–7.67 (m, 3 H), 2.81 (q, *J* = 6.9, 6.3 Hz, 2 H), 2.25 (t, *J* = 7.6 Hz, 2 H), 1.63 (p, *J* = 8.0, 7.5 Hz, 2 H). ^13^C NMR (101 MHz, DMSO-*d*_6_) δ: 174.61 (2 C=O), 167.25 (C=O), 140.24, 135.95, 135.56 (2 C), 132.15 (2 C), 128.25 (2 C), 127.78 (2 C), 124.27 (2 C), 42.66, 31.29, 25.36. HRMS (ESI), Calc. for C_18_H_17_N_2_O_6_S^+^: [M+H]^+^ 389.0802; found *m*/*z* 389.0813.

### 2.3. Synthesis and Characterization of Compounds ***3*** and ***4***

***2-((4-(N-(4-(Ethoxycarbonyl)phenyl)sulfamoyl)phenyl)carbamoyl)benzoic acid 3*** was obtained by heating ethyl 4-(4-(1,3-dioxoisoindolin-2-yl)phenylsulfonamido)benzoate **2b** (1 mmol) at 50 °C in 50% aqueous ethanol with sodium hydroxide (2 mmol) for 2 h. The reaction mixture was then cooled to room temperature and acidified with 1 N hydrochloric acid to pH 2. The precipitate was collected by filtration, washed with water and dried. Yield 84%, white powder, mp 176–178 °C. ^1^H NMR (400 MHz, DMSO-*d*_6_) δ: 13.03 (br.s, 1 H), 10.75 (s, 2 H), 7.89 (d, *J* = 6.3 Hz, 1 H), 7.85–7.76 (m, 6 H), 7.66 (t, *J* = 8.2 Hz, 1 H), 7.58 (t, *J* = 8.2 Hz, 1 H), 7.53 (d, *J* = 8.0 Hz, 1 H), 7.23 (d, *J* = 8.8 Hz, 2 H), 4.24 (q, *J* = 7.1 Hz, 2 H), 1.27 (t, *J* = 7.1 Hz, 3H). ^13^C NMR (101 MHz, DMSO-*d*_6_) δ: 168.67 (C=O), 167.80 (C=O), 165.83 (C=O), 144.29, 143.09, 139.03, 133.59, 132.57, 131.39, 131.24 (2 C), 130.35 (2 C), 128.67 (2 C), 128.41, 125.25, 119.83 (2 C), 118.77 (2 C), 61.14 (CH_2_), 14.84 (CH_3_). HRMS (ESI), Calc. for C_23_H_21_N_2_O_7_S^+^: [M+H]^+^ 469.1064; found *m/z* 469.1070. 

***2-((4-(N-(4-Carboxyphenyl)sulfamoyl)phenyl)carbamoyl)benzoic acid 4****:* The mixture (**2b+3**) or **2b** (1 mmol) in 50% aqueous ethanol with sodium hydroxide (45 mmol) was refluxed for 12 h, cooled to room temperature and acidified with 1 N hydrochloric acid to pH 2. The solid was collected by filtration, washed with water and dried to give (2-((4-(*N*-(4-carboxyphenyl)sulfamoyl)phenyl)carbamoyl)benzoic acid **4** as a white solid. Yield 82%, white powder, mp 203–205 °C. ^1^H NMR (400 MHz, DMSO-*d*_6_) δ: 12.90 (s, 2 H), 10.73 (s, 2 H), 7.89 (d, *J* = 7.7 Hz, 1 H), 7.86–7.77 (m, 6 H), 7.66 (t, *J* = 7.5 Hz, 1 H), 7.58 (t, *J* = 7.6 Hz, 1 H), 7.53 (d, *J* = 7.6 Hz, 1 H), 7.20 (d, *J* = 8.8 Hz, 2 H). ^13^C NMR (101 MHz, DMSO-*d*_6_) δ: 168.68 (C=O), 167.80 (C=O), 167.42 (C=O), 144.26, 142.78, 139.06, 133.68, 132.57, 131.40 (2 C), 130.34, 130.32, 130.29, 128.65 (2 C), 128.41, 126.13, 119.84 (2 C), 118.70 (2 C). HRMS (ESI), Calc. for C_21_H_17_N_2_O_7_S^+^: [M+H]^+^ 441.0751; found *m/z* 441.0764. 

### 2.4. Synthesis and Characterization of Compounds ***7a*** and ***7b***

The corresponding anhydride (1 mmol) was added to a solution of 4-((4-aminophenyl)sulfonamido)benzoic acid **6** (1 mmol) in glacial acetic acid (2 mL). The reaction mixture was stirred for 24 h (TLC monitoring). It was diluted with water (1 mL), filtered off, and washed with water. The resulting crude product was dried at 50 °C. A mixture of 4-(4-(3-carboxypropanamido)phenylsulfonamido)benzoic acid **6** (0.2 g, 0.5 mmol), TMSCl (0.07 g), and NMP (0.6 mL) was then heated for 2 h in a bath at 160–165 °C, cooled to room temperature, and diluted with water. The precipitate was separated by filtration, washed with water, and dried in air to obtain the target compound. These compounds were purified by flash column chromatography using CH_2_Cl_2_:MeOH (97:3) mixture as an eluent.

***4-(4-(2,5-Dioxopyrrolidin-1-yl)phenylsulfonamido)benzoic acid 7a****:* Yield 54%, beige powder, mp 243–245 °C (with decomposition). ^1^H NMR (400 MHz, DMSO-*d*_6_) δ: 12.61 (br.s, 1 H), 10.90 (s, 1 H), 7.94 (d, *J* = 8.4 Hz, 2 H), 7.81 (d, *J* = 8.5 Hz, 2 H), 7.50 (d, *J* = 8.3 Hz, 2 H), 7.22 (d, *J* = 8.5 Hz, 2 H), 2.77 (s, 4 H). ^13^C NMR (101 MHz, DMSO-*d*_6_) δ: 177.12 (2 C=O), 167.34 (C=O), 142.40, 139.21, 137.32, 131.46 (2 C), 128.36 (2 C), 128.02 (2 C), 126.47, 118.86 (2 C), 29.18 (CH_2_CH_2_). HRMS (ESI), Calc. for C_17_H_15_N_2_O_6_S^+^: [M+H]^+^ 375.0645; found *m/z* 375.0649. 

***4-(4-(1,3-Dioxo-3a,4,7,7a-tetrahydro-1H-isoindol-2(3H)-yl)phenylsulfonamido)benzoic acid 7b****:* Yield 61%, beige powder, mp 250–252 °C. ^1^H NMR (400 MHz, DMSO-*d*_6_) δ: 12.68 (br.s, 1 H), 10.89 (br.s, 1 H), 7.94 (d, *J* = 8.4 Hz, 2 H), 7.81 (d, *J* = 8.5 Hz, 2 H), 7.46 (d, *J* = 8.5 Hz, 2 H), 7.22 (d, *J* = 8.4 Hz, 2 H), 5.91 (s, 2 H), 3.32–3.27 (m, 2 H), 2.44 (d, *J* = 14.8 Hz, 2 H), 2.26 (d, *J* = 15.0 Hz, 2 H). ^13^C NMR (101 MHz, DMSO-*d*_6_) δ: 179.45 (2 C=O), 167.34 (C=O), 142.36, 139.39, 136.92, 131.46 (2 C), 128.32 (2 C), 128.15 (2 C), 126.53, 118.94 (2 C), 39.56 (2 C), 23.86 (2 C). HRMS (ESI), Calc. for C_21_H_19_N_2_O_6_S^+^: [M+H]^+^ 427.0958; found *m/z* 427.0971. 

### 2.5. Synthesis and Characterization of Compounds ***7c*** and ***7d***

The corresponding anhydride (1 mmol) was added to a solution of 4-((4-aminophenyl)sulfonamido)benzoic acid **5** (1 mmol) in glacial acetic acid (2 mL). The reaction mass was warmed to reflux and stirred for 12–24 h under reflux (TLC monitoring). It was cooled to room temperature, diluted with water (1 mL), filtered and the filter cake was washed with water. The resulting crude product was recrystallized from ethanol. 

***4-(4-(1,3-Dioxo-3a,4,7,7a-tetrahydro-1H-4,7-methanoisoindol-2(3H)-yl)phenylsulfonamido)benzoic acid 7c****:* Yield 53%, beige powder, mp 241–243 °C. ^1^H NMR (400 MHz, DMSO-*d*_6_) δ: 12.63 (br.s, 1 H), 10.91 (s, 1H, NH), 7.90 (d, *J* = 8.4 Hz, 2 H), 7.81 (d, *J* = 8.5 Hz, 2 H), 7.36 (d, *J* = 8.5 Hz, 2 H), 7.22 (d, *J* = 8.6 Hz, 2 H), 6.20 (s, 2 H), 3.50 (s, 2 H), 1.59 (s, 2 H). ^13^C NMR (101 MHz, DMSO-*d*_6_) δ: 176.91 (2 C=O), 167.33 (C=O), 142.37, 139.40, 136.69, 135.22 (2 C), 131.49 (2 C), 128.43 (2 C), 128.07 (2 C), 126.44, 118.75 (HC=CH), 52.41, 46.22 (2 C), 45.55 (2 C). HRMS (ESI), Calc. for C_22_H_19_N_2_O_6_S^+^: [M+H]^+^ 439.0958; found *m/z* 439.0967. 

***4-(4-(1,3-Dioxohexahydro-1H-4,7-methanoisoindol-2(3H)-yl)phenylsulfonamido)benzoic acid 7d****:* Yield 66%, beige powder, mp 265–267 °C. ^1^H NMR (400 MHz, DMSO-d6) δ: 12.68 (br.s, 1 H), 10.92 (br.s, 1 H), 7.95 (d, *J* = 8.6 Hz, 2 H), 7.82 (d, *J* = 8.7 Hz, 2 H), 7.48 (d, *J* = 8.6 Hz, 2 H), 7.24 (d, *J* = 8.6 Hz, 2 H), 3.26 (s, *J* = 2.5 Hz, 2 H), 2.66 (s, 2 H), 1.66 (d, *J* = 9.9 Hz, 1 H), 1.60–1.50 (m, 3 H), 1.25 (d, *J* = 8.5 Hz, 2 H). ^13^C NMR (101 MHz, DMSO-*d*_6_) δ: 177.56 (2 C=O), 167.33 (C=O), 142.38, 139.59, 136.80, 131.50 (2 C), 128.61 (2 C), 128.18 (2 C), 126.47, 118.80 (2 C), 49.15, 42.14, 41.11, 39.61, 25.08. HRMS (ESI), Calc. for C_22_H_21_N_2_O_6_S^+^: [M+H]^+^ 441.1115; found *m/z* 441.1124.

### 2.6. Synthesis and Characterization of Compound ***7e***

Anhydrous zinc acetate (1 mmol) and compound **5** (1 mmol) were added under nitrogen to a mixture of naphthalene-1,8-dicarboxylic anhydride (1 mmol) and 10 mL of anhydrous N-methylpyrrolidone. Next, the mixture was heated to 160 °C and stirred at this temperature for 15 h. After cooling to room temperature, the product was precipitated by adding 5 mL of 5% hydrochloric acid, filtered off, washed first with 5% hydrochloric acid and then with hot water, and dried. The product was obtained as a brown solid, which corresponds to a yield of 94%. The resulting crude product was purified by short-path chromatography on silica gel using ethyl acetate as an eluent.

***4-(4-(1,3-Dioxo-1H-benzo[de]isoquinolin-2(3H)-yl)phenylsulfonamido)benzoic acid 7e:*** Yield 58%, beige powder, mp 284–286 °C. ^1^H NMR (400 MHz, DMSO-*d*_6_) δ: 12.74 (br.s, 1 H), 10.98 (s, 1 H), 8.53–8.48 (m, 4 H), 7.99 (d, *J* = 8.4 Hz, 2 H), 7.93–7.82 (m, 4 H), 7.64 (d, *J* = 8.4 Hz, 2 H), 7.29 (d, *J* = 8.5 Hz, 2 H). ^13^C NMR (101 MHz, DMSO-*d*_6_) δ: 167.37 (C=O), 164.13 (2 C=O), 142.50, 141.01, 139.89, 135.32 (2 C), 132.12, 131.49 (2 C), 131.19 (2 C), 130.85, 130.00, 128.54, 127.99, 127.93 (2 C), 126.45, 123.10 (2 C), 120.01, 118.84 (2 C). HRMS (ESI), Calc. for C_25_H_17_N_2_O_6_S^+^: [M+H]^+^ 473.0802; found *m/z* 473.0815. 

### 2.7. Viruses and Cells

Coxsackievirus B3 (CVB3, strain Nancy) was obtained from the collection of viruses of the Pasteur Institute (St. Petersburg, Russia). The permissive cell line Vero (ATCC #CCL-81) was used in the study. Infectious titers (in a 50%-tissue culture infection dose, TCID50) were determined in Vero cells for CVB3 by endpoint dilution assay using the following procedure. Permissive cells were seeded into 96-wells plates in Eagles minimal essential medium (MEM) supplemented with 5% fetal bovine serum (FBS). After 24 h, the medium was aspirated, the wells were washed with saline, fresh MEM without FBS was added to the wells and the cells were infected with serial tenfold dilutions of virus stocks (100 µL per well, 4 wells for each dilution). The plates were incubated at +37 °C in 5% CO_2_ and observed daily for cytopathic effect (CPE). After 72 h the viral titer was calculated in TCID50 using the method of Spearmen-Carber.

### 2.8. Cytoprotection Assay

Vero cells were seeded in 96-well plates in 24 h before the assay. On the assay day, the cells were washed with saline and tested compounds in appropriate concentrations (300–3 μg/mL) in MEM without FBS were added to cells. No compounds were added to virus control wells. The plates were incubated for 1 h at 37 °C at 5% CO_2_. The cells were then infected with CVB3 Nancy (m.o.i range of 1–0.001) and incubated for 72 h at 37 °C at 5% CO_2_. No virus was added to the cytotoxicity control wells. Cell viability was then assessed using an MTT test with the following procedure. The cells were washed with saline, and a solution of 3-(4,5-dimethylthiazolyl-2) 2,5-diphenyltetrazolium bromide (ICN Biochemicals Inc., Aurora, OH, USA) (0.5 µg/mL) in MEM was added to the wells (100 µL per well). After 2 h of incubation at 37 °C in 5% CO_2_, the supernatant from wells was discarded, and the formazan residue was dissolved in DMSO (100 µL per well). The optical density of cells was then measured on a Multiskan multifunctional reader (Thermo Fisher Scientific, Shanghai, China) at a wavelength of 540 nm. 

The cytoprotective activity of compounds was considered to be their ability to increase the values of OD compared to control wells (using only a virus, without drugs). Based on the results obtained, the values of IC_50_, i.e., the concentration of compounds that result in 50% cells protection were calculated for m.o.i. 0.001 using GraphPad Prism 6.01 software.

### 2.9. Cytotoxicity Assay

The microtetrazolium test (MTT) was used to study the cytotoxicity of the compounds. The permissive cell line Vero was seeded in 96-well plates in Eagles minimal essential medium (MEM) supplemented with 10% FBS. After 24 h, the media were removed, and the wells were washed with saline. Compounds were dissolved in DMSO, and a series of two-fold dilutions of each compound in MEM without FBS were prepared and added to the cells in triplicates. Cells were incubated for 24 h at 37 °C in 5% CO_2_. The MTT was then performed as described above. Next, the optical density of cells was measured on a Multiskan multifunctional reader (Thermo Fisher Scientific, Shanghai, China) at a wavelength of 540 nm and plotted against the concentration of the compounds to generate the dose–response curve. The 50% cytotoxic dose (CC_50_) of each compound (i.e., the compound concentration that causes the death of 50% of cells in a culture, or decreases the optical density twice, as compared to the control wells) was calculated using a four-parameter logistic nonlinear regression model (GraphPad Prism 6.01).

### 2.10. Antiviral Activity Determination

The antiviral activity of the compounds was evaluated using viral yield reduction assay. The respective permissive cell line Vero was seeded in MEM supplemented with 5% FBS in 24-well plates. When the cell confluence reached 100%, the compounds tested were dissolved in DMSO, and a series of three-fold dilutions of each compound in MEM without FBS was added to the cells, and incubated at 37 °C in 5% CO_2_. After 1 h, the media were discarded; equal volumes of fresh serial dilutions of each compound and viral suspension in MEM without FBS at MOI 0.0 were added. In control wells, only MEM without FBS was added. No compounds were added in the virus control wells. The plates were incubated at 4 °C for 1 h. The unbound virus was then washed away and, again, three-fold dilutions of each compound (the final concentration 50–0.6 µg/mL) in MEM without FBS were added to the wells. After 24 h of incubation at 37 °C in 5% CO_2_, the infectious titer of viral progeny (in TCID50) for each compound concentration, cell control, and virus control wells were determined in permissive cell lines by endpoint dilution assay as described above. 

The titer of virus progeny was plotted against log concentration of the compounds tested to generate the dose–response curve. The 50% inhibition concentration (IC_50_) of each compound tested (i.e., the compound concentration that decreases the infectious viral progeny titer twice, as compared to the control wells) was calculated using a four-parameter logistic nonlinear regression model (GraphPad Prism 6.01).

Selectivity index (SI) was calculated for each compound tested as a ratio of CC_50_ to IC_50_ values.

### 2.11. Thermostability Assay

CVB3 Nancy was pre-incubated with the compounds tested or an equal volume of MEM (virus control) for 30 min at +37 °C in sterile thin-walled 200 μL PCR-tubes (7 tubes per each treatment condition) in BioRad CFX PCR-machine. The thermal gradient of 37–55 °C for 2 min was then applied, followed by rapid cooling to 4 °C. Subsequently, the infectious virus load at each thermal condition for each compound tested was quantified by end-point dilution assay.

### 2.12. Statistics

All *in vitro* experiments were repeated three times. The results were analyzed using GraphPad Prism 6.01.

## 3. Results

### 3.1. Synthesis of Antiviral Compounds

**Scheme 1:**
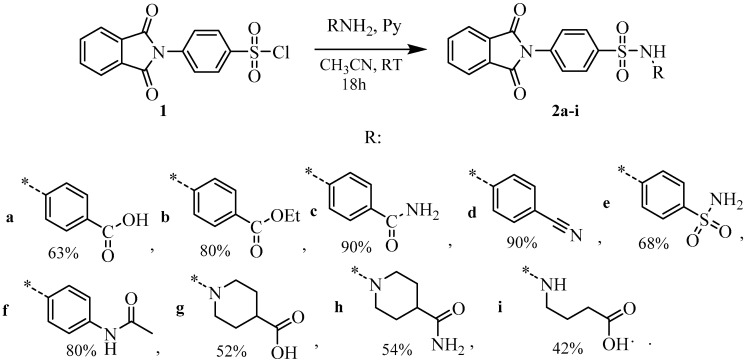
Synthesis of sulfonamides **2a**–**i**. The * sign denotes the attachment position of the substituent.

At the first stage, we successfully reproduced the scheme for the synthesis of the lead-compound **2a** [14] and tried to modify the binding groups and their effect on the antiviral properties of obtained compounds (Scheme 1). Starting phenylsulfonyl chloride **1** was obtained by treating *N*-phenylphthalimide with chlorosulfonic acid in the presence of PCl_5,_ according to the method [15]. The sulfonyl chloride **1** obtained with a 55% yield was reacted with amines in an acetonitrile medium in the presence of pyridine.

**Scheme 2:**
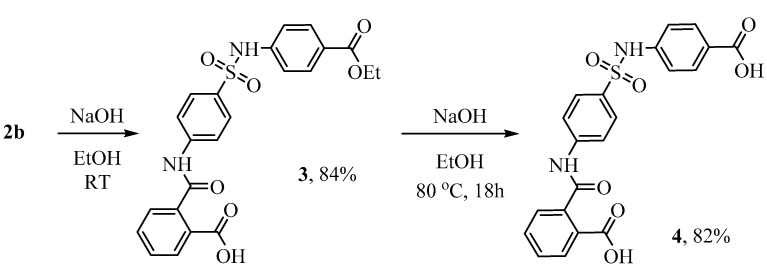
Hydrolysis of **2b** in the basic conditions.

An attempt to convert compound **2b** into the lead-compound **2a** via base hydrolysis failed (Scheme 2).

It was shown that compound **2b** in a base medium in the temperature range from 0 to 50 °C undergoes imide ring opening with the remaining ester function. It has been established that the hydrolysis of ester moiety requires prolonged reflux in ethanol with at least a threefold excess of sodium hydroxide (Scheme 2). Both compounds (**3** and **4**) were isolated and fully characterized using NMR spectroscopy and HR-mass spectrometry. The presence of two new proton signals (at 10.75 and 13.03 ppm) in ^1^H NMR spectrum of **3** in comparison with ester **2b** is evidence to the phthalimide ring opening. Characteristics of aromatic protons and carbons also changed. Particularly, the number of signals increased for both carbons and protons (see Section 2.2 and Section 2.3, as well as Supplementary Materials).

**Scheme 3:**
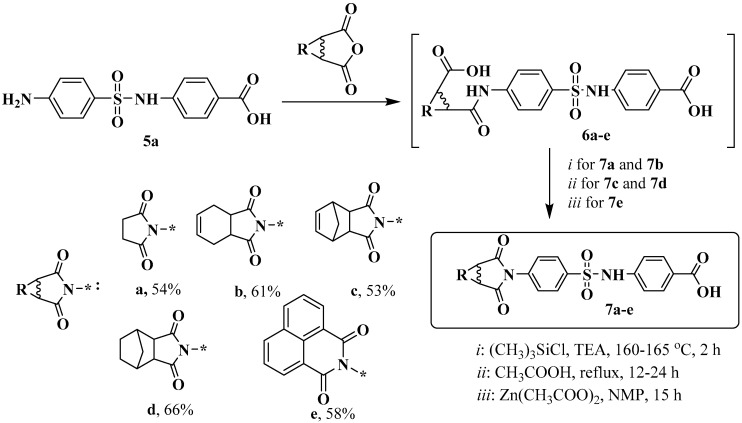
Synthesis of imides **7a**–**e**. The * sign denotes the attachment position of the substituent.

In addition, in order to change the lipophilic properties and the spatial structure of the ligand periphery, we replaced the phthalimide group with fragments of naphthalic anhydride **7e** and anhydrides of cycloalkanedicarboxylic acids **7a**–**d** (Scheme 3).

To perform this modification, the starting compound **5** was used, which was obtained with a total yield of 44% according to the known method [15]. The method was based on commercially available 4-*N*-acetaminophenylsulfonyl chloride, which was reacted with aminobenzoic acid in the presence of pyridine, followed by hydrolysis of the amide group with sodium hydroxide. Amino derivative **5** had low nucleophilic properties and entered into cyclization reactions to form compounds **7** with difficulty. Attempts to obtain imides **7** directly from amine **5** by prolonged refluxing of the reagents in acetic acid gave a positive result only in the case of compounds **7c** and **7d**. Compound **7e** was successfully synthesized by heating it in *N*-methylpyrollidone in the presence of zinc acetate, according to the method [18]. Moderate yields of products **7a** and **7b** were obtained from compounds **6** by the action of trimethylchlorosilane in dimethylformamide, according to the method [19].

As a result, the 15 new 4-substituted sulfonamidobenzoic acids and its surrogates were synthesized using the routes described above. 

### 3.2. Results of In Vitro Biological Studies 

At the beginning of our study, cytoprotection properties of compounds against the pleconaril-resistant strain Coxsackievirus B3 Nancy [20] were determined using a well-known cytoprotection assay. Cytoprotection assay allows the evaluation of cell survival depending on the concentration of tested compounds after virus infection. Cell survival is assessed by MTT assay based on the measurement of cell respiration. Compound **2a** was used for the comparison. The results of the cytoprotection assay are presented in Table 1.

As shown in Table 1, 7 compounds out of 15 (46% of total tested) demonstrated substantial cytoprotective activity with an SI that was much more than 10. These compounds were selected for further analysis. Compounds **2f** and **3** were the most toxic compounds, while for compounds **7c**, **2h**, and **4** value of CC_50_ exceeded the maximum concentration used in the assay (the same as for the reference compound **2a**).

To confirm virus-inhibition activity for selected compounds we then performed a viral yield reduction test. As described above, this test was applied for measuring the titer of viral progeny in the supernatant of the infected culture depending on the compound concentration. Therefore, it is possible to evaluate the direct antiviral activity of the compounds. The results of the viral yield reduction assay are summarized in the Table 2 below. Compound **2a** was used for comparison.

According to the results of the viral yield assay, two compounds—**7a** and **4**—demonstrated potent inhibiting properties with IC_50_ lower than that of the reference compound, **2a**. They all had comparable cytotoxicity at the 24 h time point, but the SI’s of **7a** and **4** were higher than of those of the reference compound (**2a**). 

Next, we checked whether the most active candidates (**4** and **7a**) belonged to the capsid binder group in the thermostability assay. Capsid thermostability assay is one of the approaches to determine the ability of the compound to bind viral particles. It is known that capsid binders (for example, well-known first in-class capsid binder pleconaril and its derivatives) directly interact with the capsid of non-enveloped viruses and stabilize its structure, preventing the virus from entering the host cell and increasing their stability to heat inactivation. 

**Figure 2:**
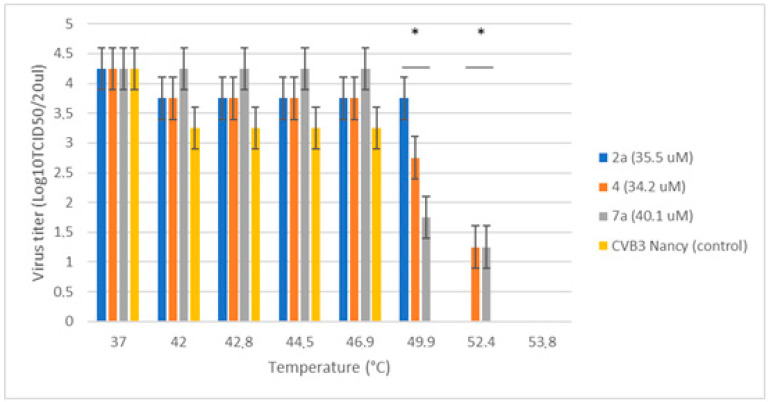
Thermostabilizing properties of **7a** and **4** in comparison to **2a**. Values are the mean ± SD of four independent experiments. The legend shows the concentration of each compound tested. The asterisk indicates the significance of the difference in virus titer for **2a**, **4**, **7a** at 49.9 °C and 52.4 °C relative to the virus control, *p* < 0.05 by Mann–Whitney U-test.

CVB3 Nancy was pre-incubated with **4, 7a** or **2a** at concentrations greatly exceeding their IC_50_ values to allow for strong binding [14,21]. After that, the viral suspension was gradually heated and rapidly cooled down. The infectious titer of CVB3 corresponding to each treatment condition is presented in Figure 2 below.

It can be seen that in no treatment virus control CVB3 Nancy virus titer gradually decreased with increasing temperature. No infectious particles were determined at 49.9 °C and above. In contrast to the virus control sample, upon addition of **4**, **7a** and **2a, the** heat inactivation of CVB3 changed to higher temperatures, suggesting that all the compounds tested stabilize the viral capsid and protect the virus from heat degradation. Both **7a** and **4** were able to preserve the infectivity of CVB3 to the same extent (to 52.4 °C), which slightly exceeded the activity of the reference compound (**2a**). Thus, we confirm that these new derivatives of *N*-sulfonamidobenzoic acid also belong to the capsid binder class of enterovirus inhibitors.

### 3.3. Structure-Activity-Relationship

It was shown that the hydroxyl fragment of the carboxyl groups of aminobenzoic acid is primarily responsible for ligand-receptor binding. Attempts to change the lipophilicity of this fragment by esterification of the carboxyl group (**2b**) or by the formation of amide (**2c**) and nitrile (**2d**), sulfonamide (**2e**) and acetamide (**2e**) derivatives negatively affected the antiviral activity of the molecules. In some cases, antiviral activity completely disappeared.

Attempts to replace the terminal aminobenzoic acid while retaining the carboxyl function (examples **2g**, **2h**, **2i**) with linear and cycloaliphatic fragments that are more conformationally mobile do not increase the activity of the target ligands. Taking into account the propensity of the imide fragment to hydrolysis reactions, including in physiological media, we synthesized non-imide analogs of compound **2a** and gave compound **4** as a potent ligand (first hit). In addition, the phthalimide moiety was replaced by several cyclic aliphatic imides. Although in most cases the inhibitory activity was lower in comparison with **2a**, succinimide **7a** demonstrated good potency and was recognized as the second hit compound.

In this regard, it should be concluded that in the course of the work carried out on the medicinal-chemistry modification of the lead-compound, two new, highly active compounds were identified that are superior in activity to compound **2** against the Coxsackievirus B3 Nancy. The risks of degradation of the lead compound upon contact with hydrolytic media with the formation of metabolites (**4**), which also have an antiviral effect, are shown. In this regard, changing the method of delivery of this innovative substance may be recommended. 

## 4. Discussion

Enteroviral infection is a serious threat to public health as it affects millions of people of different age groups. Despite the wide distribution of enteroviruses and the economic damage due to cases of temporary disability caused by enterovirus infection, no approved antivirals are available to treat non-polio enteroviral infections, and the vaccination option is available only for polioviruses and EV71. Recently, a new class of capsid binders was described (derivatives of 4-substituted sulfonamidobenzoic acid), which inhibit a variety of enteroviruses by occupying a positively charged surface depression at a conserved VP1–VP3 interprotomer interface in the viral capsid [14].

In this study, we prepared 15 novel compounds bearing 4-substitutedsulfonyl-)4-aminobenzoic acid and their surrogates, which are characterized in antiviral assays *in vitro*.

We identified two hit-compounds (2-((4-(*N*-(4-carboxyphenyl)sulfamoyl)phenyl)carbamoyl)benzoic acid (**4**) and 4-(4-(2,5-dioxopyrrolidin-1-yl)phenylsulfonamido)benzoic acid (**7a**) from the initial set with IC_50_ values of 4.29 and 4.22 μM *in vitro* antiviral activity, and characterized them as a new high-affinity capsid binders that act on pleconaril-resistant Coxsackievirus B3 Nancy strains at a low μM concentration range.

Structural features of the pharmacophore fragment of the molecule responsible for the binding of the ligand to the viral capsid were identified. It was shown that the hydroxyl fragment of the carboxyl group of aminobenzoic acid is primarily responsible for ligand–receptor binding. Esterification, amidation, or replacement of the carboxyl function with a nitrile, sulfonamide, or acetamide moiety, or replacement of aminobenzoic acid with amino acids of a similar structure—all led to the disappearance of antiviral activity.

We proved that selected lead-compounds belong to the capsid binder group of inhibitors, as their prototype molecule does. It is widely accepted that viral capsid is undeniably an attractive target for the development of direct-acting inhibitors that can interfere with viral entry. Unfortunately, most capsid binders have a major disadvantage—an obvious low barrier to resistance emergence due to mutations in amino acid residues either directly involved in the formation of the hydrophobic binding site or outside the binding pocket [22,23]. Combinational therapy is believed to be an option to overcome this problem. Cocktails of antivirals are used to target multiple viruses and mitigate the development of antiviral drug resistance or delay its emergence [24,25,26]. The application of synergistic drug combinations allows the concentration of antivirals to be reduced, which in turn could decrease the side effects of individual drugs. Therefore, new drugs and their combinations need to be identified to inhibit different enteroviruses. The results of the present study, where we identified novel capsid-binding small molecules with improved activity and low toxicity *in vitro*, can be used to develop such therapies.

## 5. Conclusions

Currently, dozens of antiviral therapies are at different stages of development, but there are no approved drugs available for the effective management of severe enteroviral infections. Here, new 4-substituted sulfonamidobenzoic acids have shown high *in vitro* antiviral activity against enteroviruses group B, realizing their antiviral potential by binding to the virus capsid and preventing infection of the permissive cell culture. The hydroxyl fragment of the carboxyl group of aminobenzoic acid is primarily responsible for ligand–receptor binding. Changing the carboxyl function or replacing the aminobenzoic acid with amino acids of a similar structure is detrimental to antiviral activity. In the absence of registered drugs on the market for the prevention and treatment of non-polio enterovirus infections, the results obtained serve as the basis for the further development of this group of compounds.

## Figures and Tables

**Table 1:** Results of cytoprotection assay of synthesized 4-substituted sulfonamidobenzoic acid derivatives against CVB3 Nancy.

Compound ID	CC_50_, (μM) *	EC_50_, (μM) **	SI
**2a**	>710.20	<8.76	>81.1
**2b**	39.96 ± 4.12	>443.98	<1
**2c**	472.21 ± 38.17	>474.57	<1
**2d**	624.66 ± 57.47	743.64 ± 67.32	<1
**2e**	437.17 ± 35.12	>437.17	<1
**2f**	181.42 ± 19.21	>688.93	<1
**2g**	>228.31	12.33 ± 2.07	>18.5
**2h**	>725.58	>725.58	>1
**2i**	283.21 ± 31.09	<28.58	>10
**3**	>640.35	>426.90	>1
**4**	>227.27	13.21 ± 1.49	>17.2
**7a**	344.58 ± 32.16	<9.88	>34.8
**7b**	473.67 ± 38.52	<8.67	>54.6
**7c**	>684.21	<8.44	>81.1
**7d**	681.08 ± 54.82	<8.39	>81.1
**7e**	531.24 ± 48.36	>634.95	<1

* CC_50_ is the cytotoxic concentration resulting in the death of 50% of the cells; CC_50_ was evaluated after 72 h of incubation of Vero cells with the compound only. ** EC_50_ is the 50% cytoprotective concentration leading to 50% cytoprotection after infection of cells with CVB3 at m.o.i 0.001; c SI is the selectivity index, the ratio of CC_50_/IC_50_. Compound **2a** was used as a reference (positive control). The data presented were obtained from three independent experiments and the values for CC_50_ and EC_50_ are mean ± SD.

**Table 2:** Virus-inhibiting properties of selected compounds against Coxsackievirus B3 (Nancy) *in vitro*.

Compound	CC_50_, (μM) *	IC_50_, (μM) **	SI
**2a**	>1183.68	5.54 ± 0.89	>213
**2i**	1261.59 ± 110.34	37.85 ± 1.08	33
**4**	>1135.25	4.29 ± 1.79	>264
**7a**	>1335.58	4.22 ± 0.35	>316
**7b**	933.29 ± 75.13	11.14 ± 0.16	33
**7c**	>1140.36	19.55 ± 0.52	>58
**7d**	>1135.13	19.61 ± 0.49	>58

MOI = 0.01. * CC_50_ is the cytotoxic concentration resulting in the death of 50% of the cells; CC_50_ was evaluated after 24 h of incubation of Vero cells with the compound only. ** IC_50_ is the 50% virus-inhibiting concentration leading to 50% inhibition of virus replication after infection of cells with CVB3 at m.o.i 0.01; c SI is the selectivity index, the ratio of CC_50_/IC_50_. The data presented were obtained from three independent experiments and the values for CC_50_ and IC_50_ are mean ± SD of the experiment.

## Data Availability

Not applicable.

## Supplementary Materials

The following supporting information can be downloaded at: https://www.mdpi.com/article/10.3390/life12111832/s1. Copies of ^1^H and ^13^C NMR spectra.

## Sample Availability

Samples of the compounds are not available from the authors.
