# Supplementary material for: Design of 4-Substituted Sulfonamidobenzoic Acid Derivatives Targeting Coxsackievirus B3
Source: Life (Basel). 2022 Nov 9;12(11):1832. doi: 10.3390/life12111832 (PMC9694965; doi:10.3390/life12111832)

^{1}H and ^{13}C NMR spectra of 4-(4-(1,3-dioxoisoindolin-2-yl)phenylsulfonamido)benzoic acid **2a**

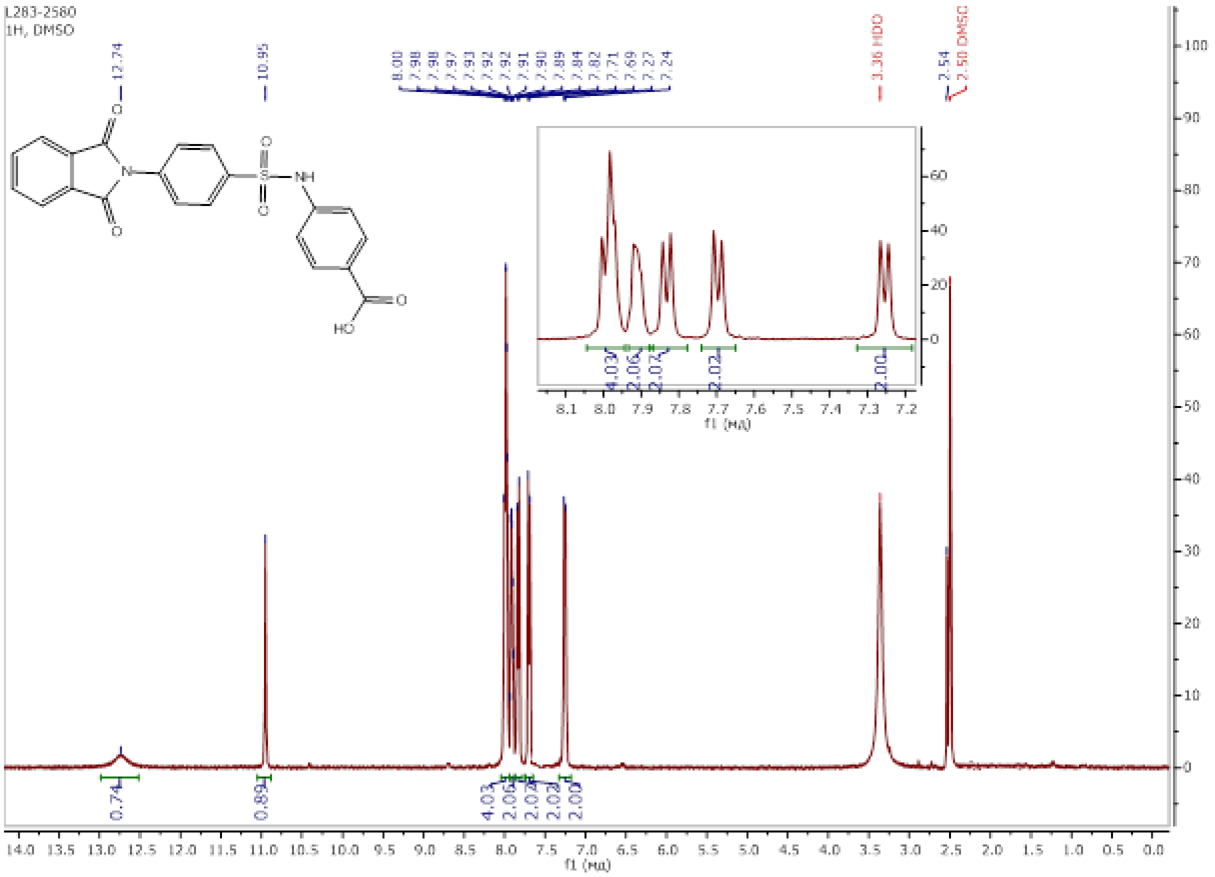

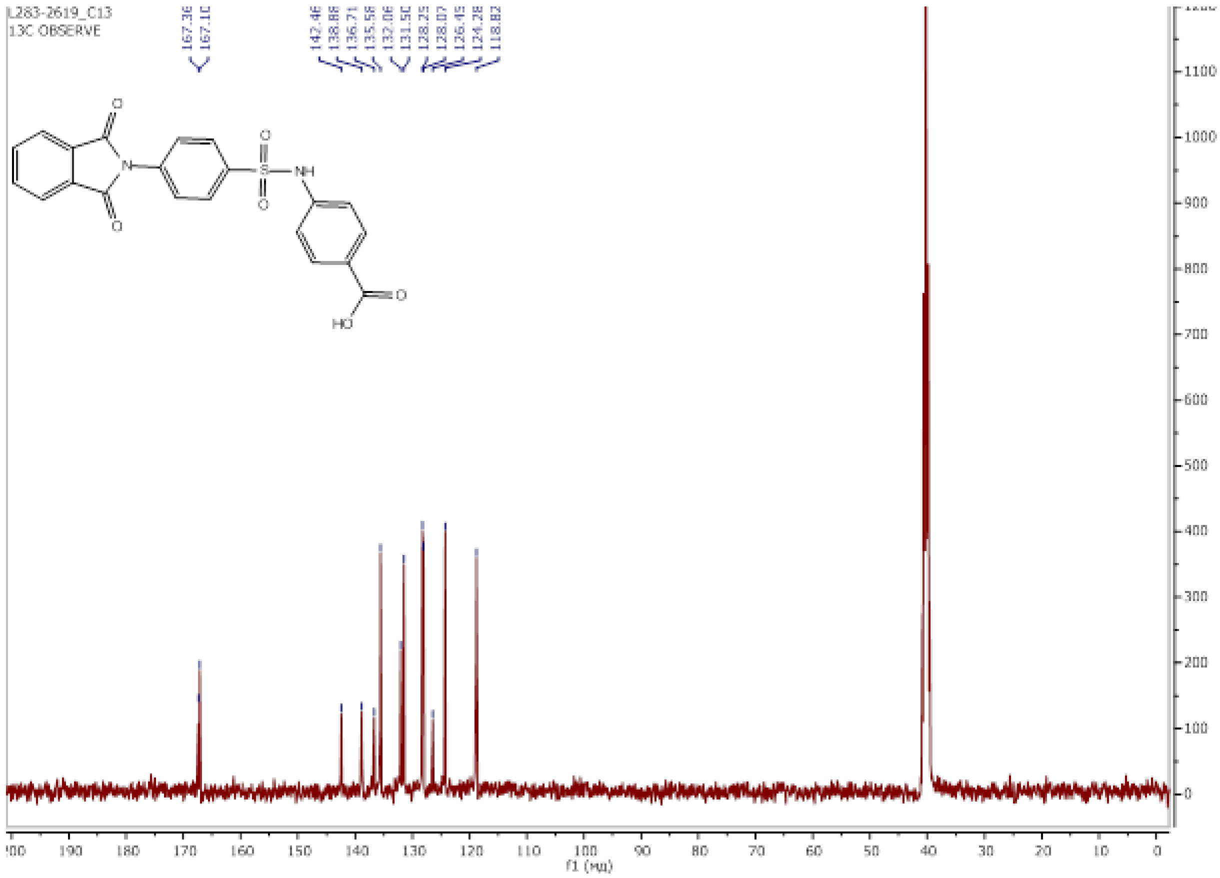

^{1}H and ^{13}C NMR spectra of ethyl 4-(4-(1,3-dioxoisoindolin-2-yl)phenylsulfonamido)benzoate **2b**

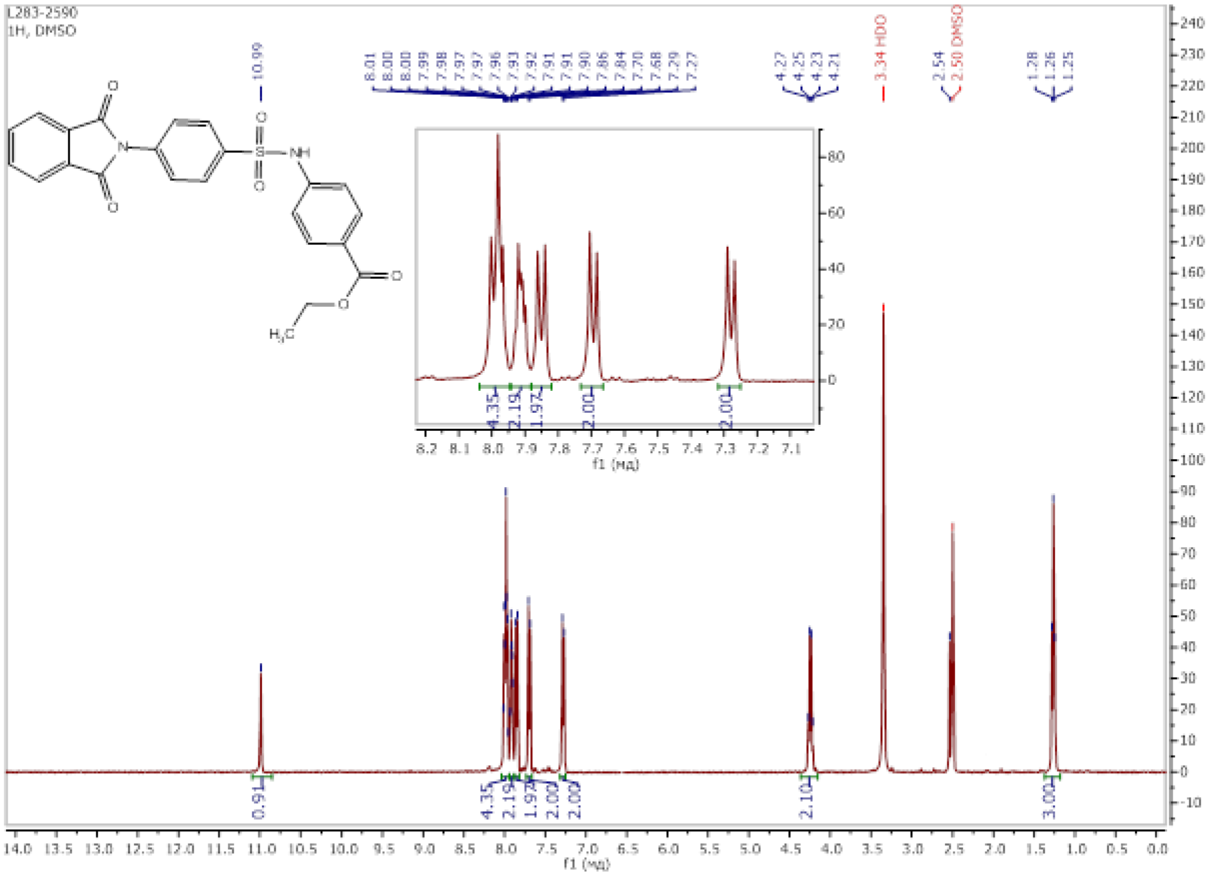

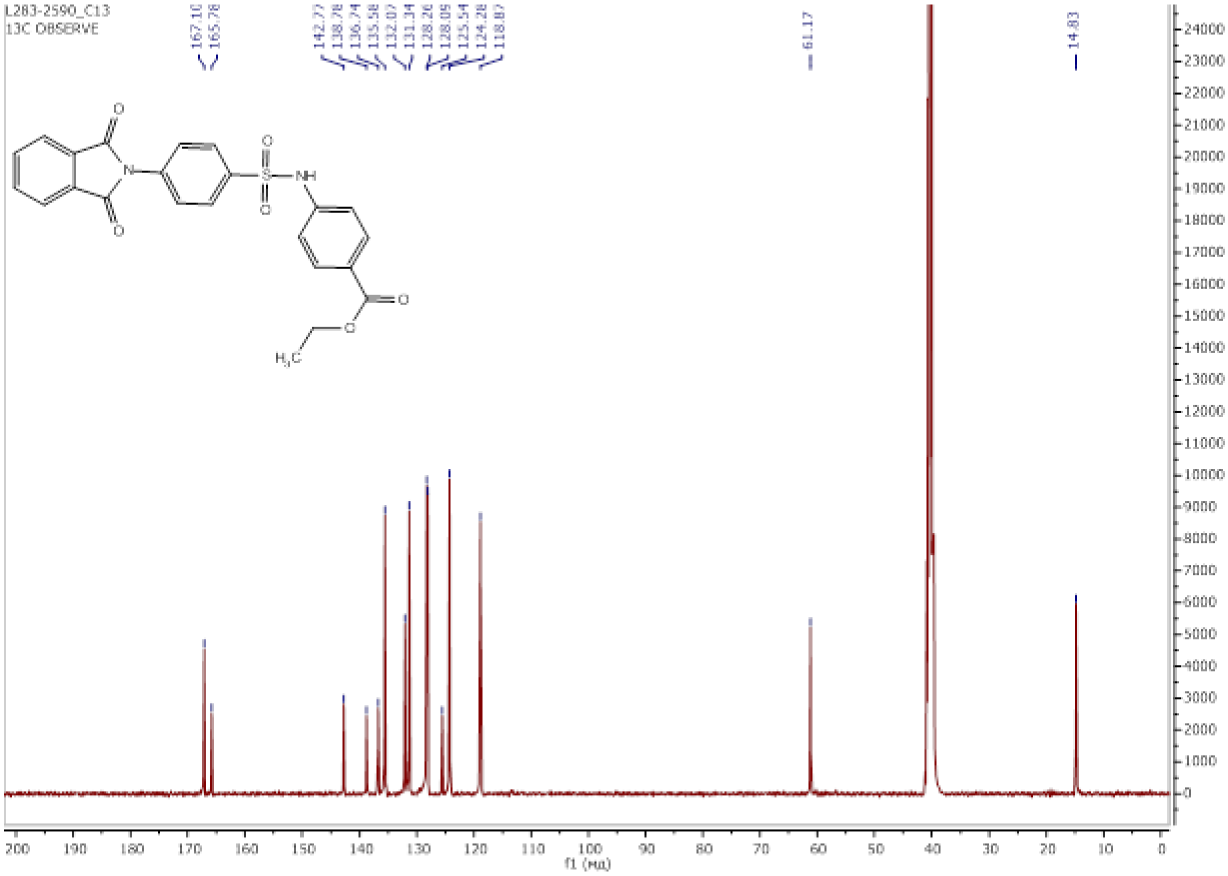

^{1}H and ^{13}C NMR spectra of 4-(4-(1,3-dioxoisoindolin-2-yl)phenylsulfonamido)benzamide **2c**

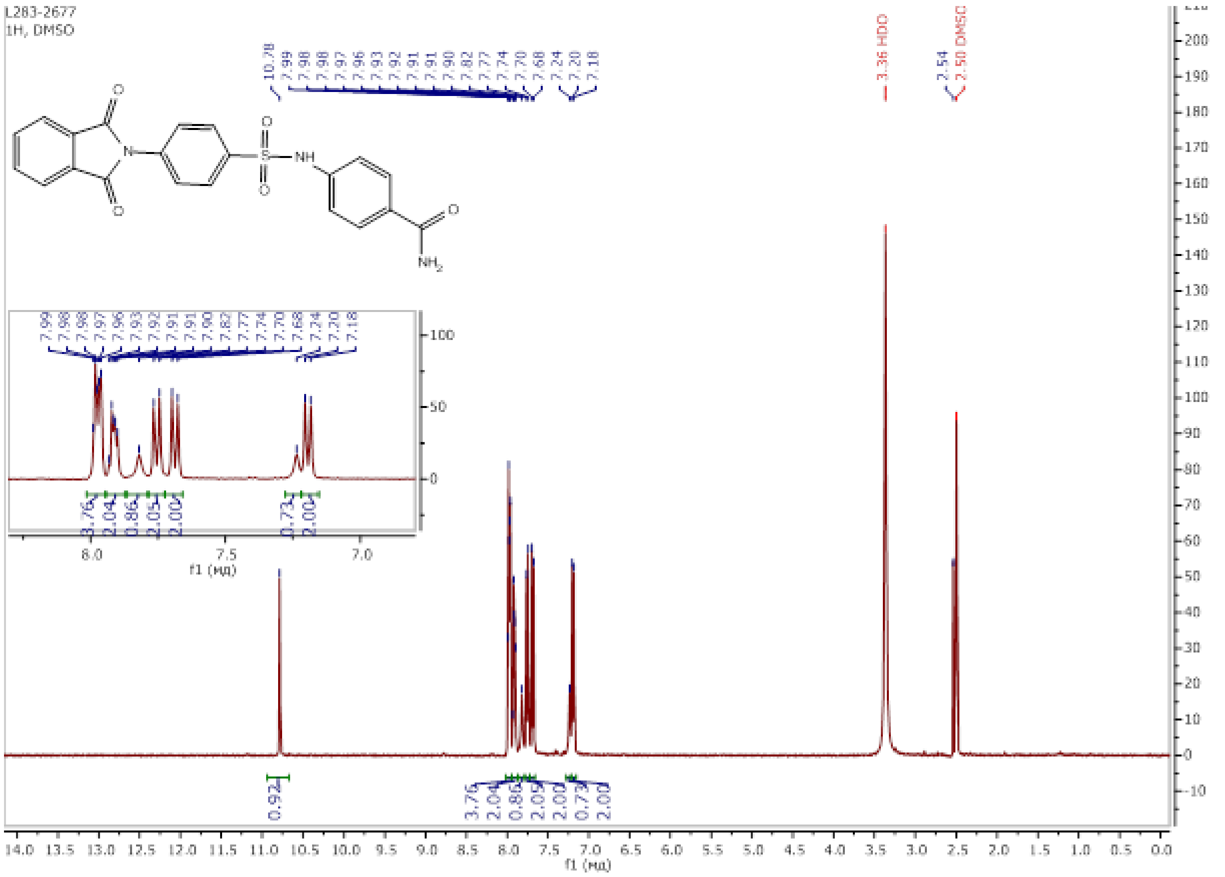

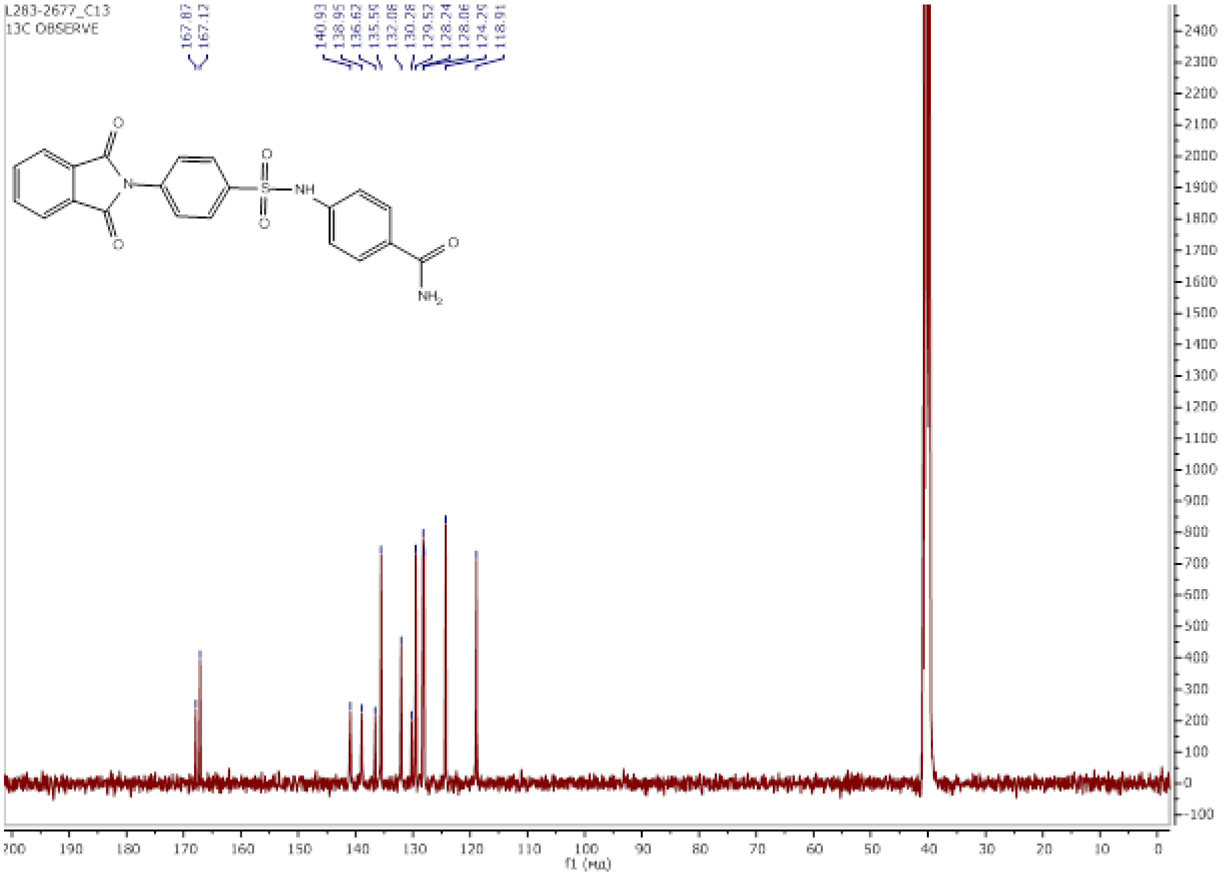

^{1}H and ^{13}C NMR spectra of *N*-(4-cyanophenyl)-4-(1,3-dioxoisoindolin-2-yl)benzenesulfonamide **2d**

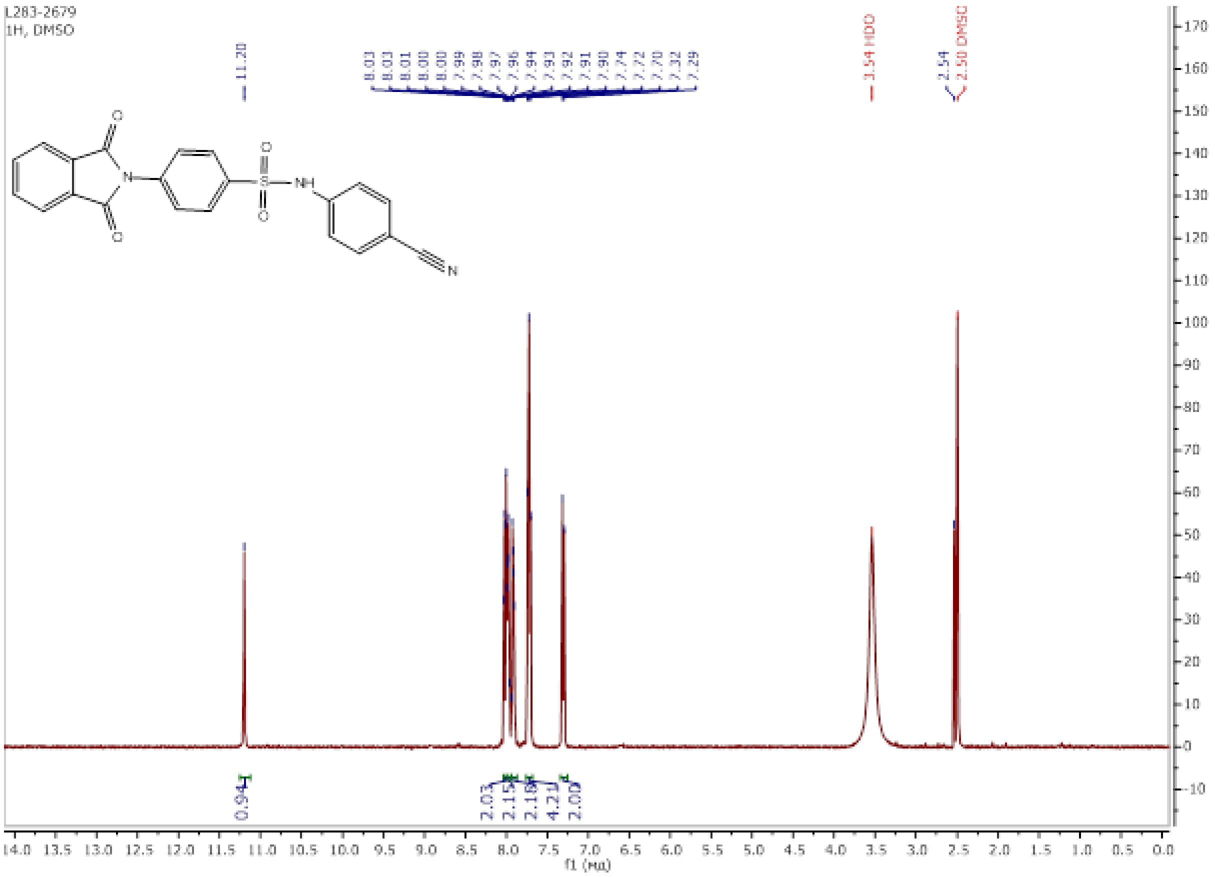

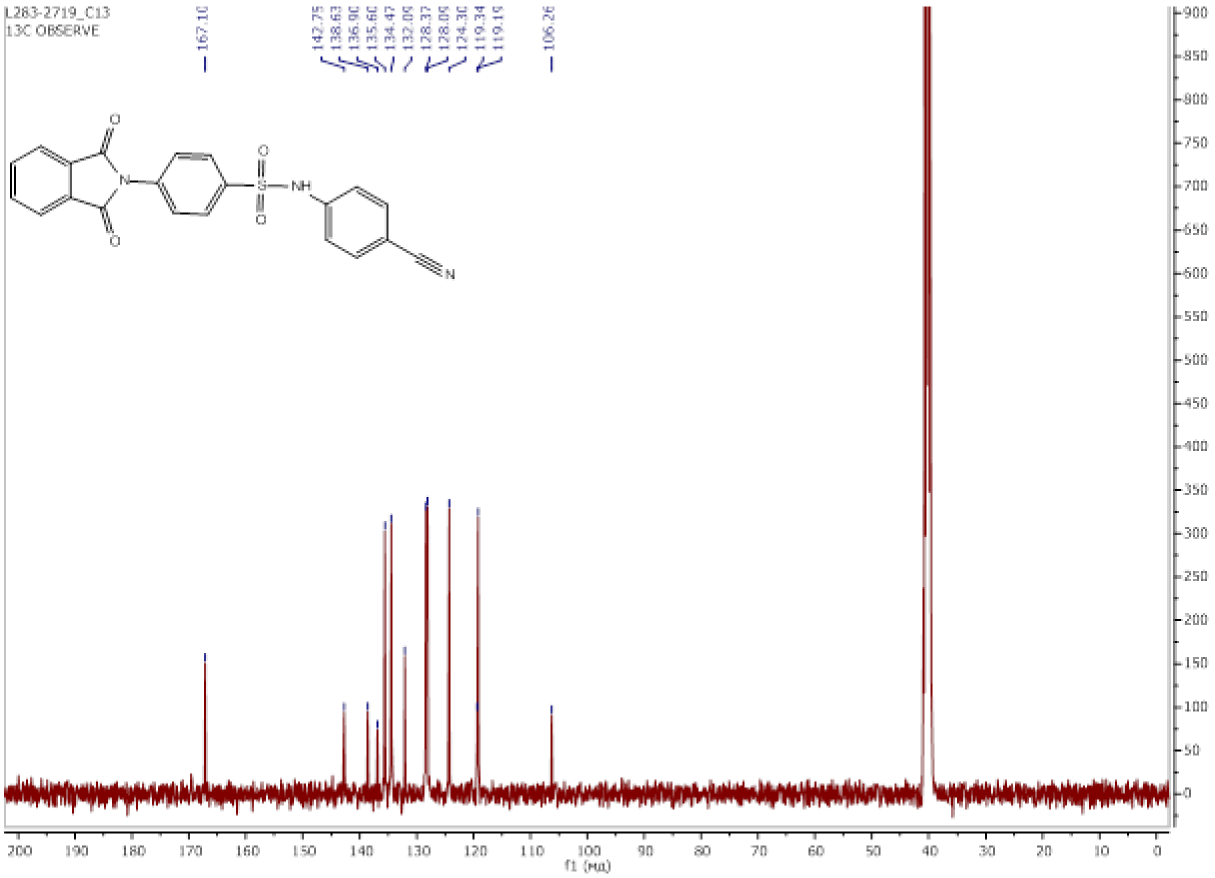

^{1}H and ^{13}C NMR spectra of 4-(1,3-dioxoisoindolin-2-yl)-N-(4-sulfamoylphenyl)benzenesulfonamide **2e**

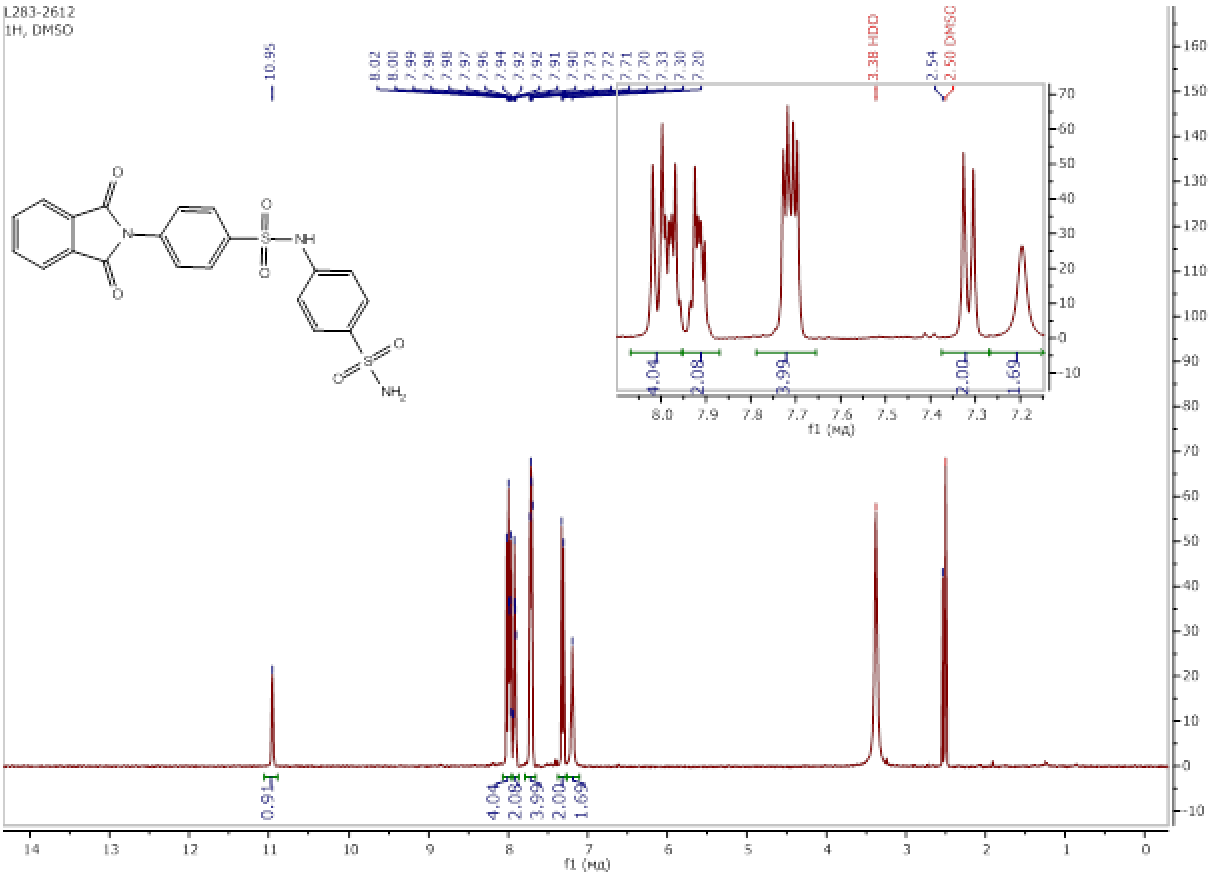

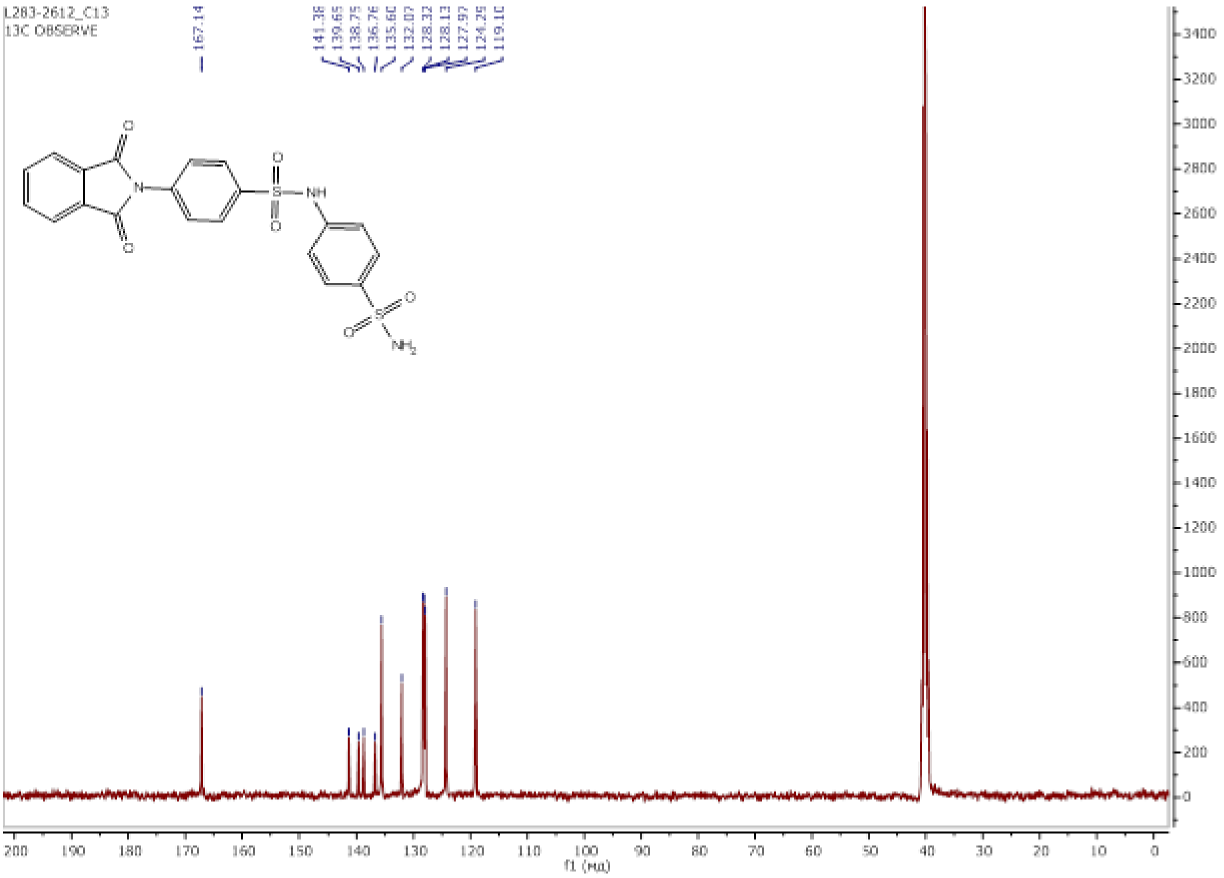

^{1}H and ^{13}C NMR spectra of *N*-(4-(4-(1,3-dioxoisoindolin-2-yl)phenylsulfonamido)phenyl)acetamide **2f**

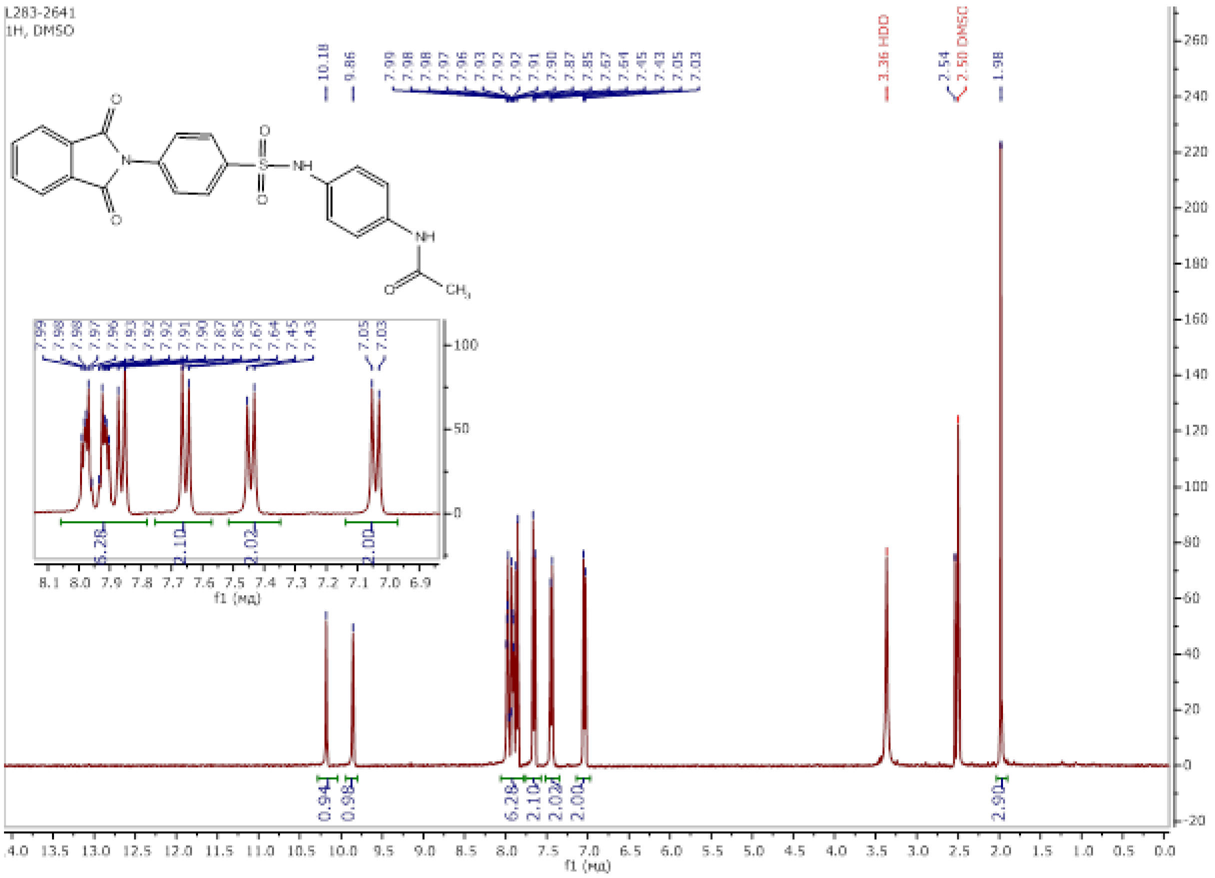

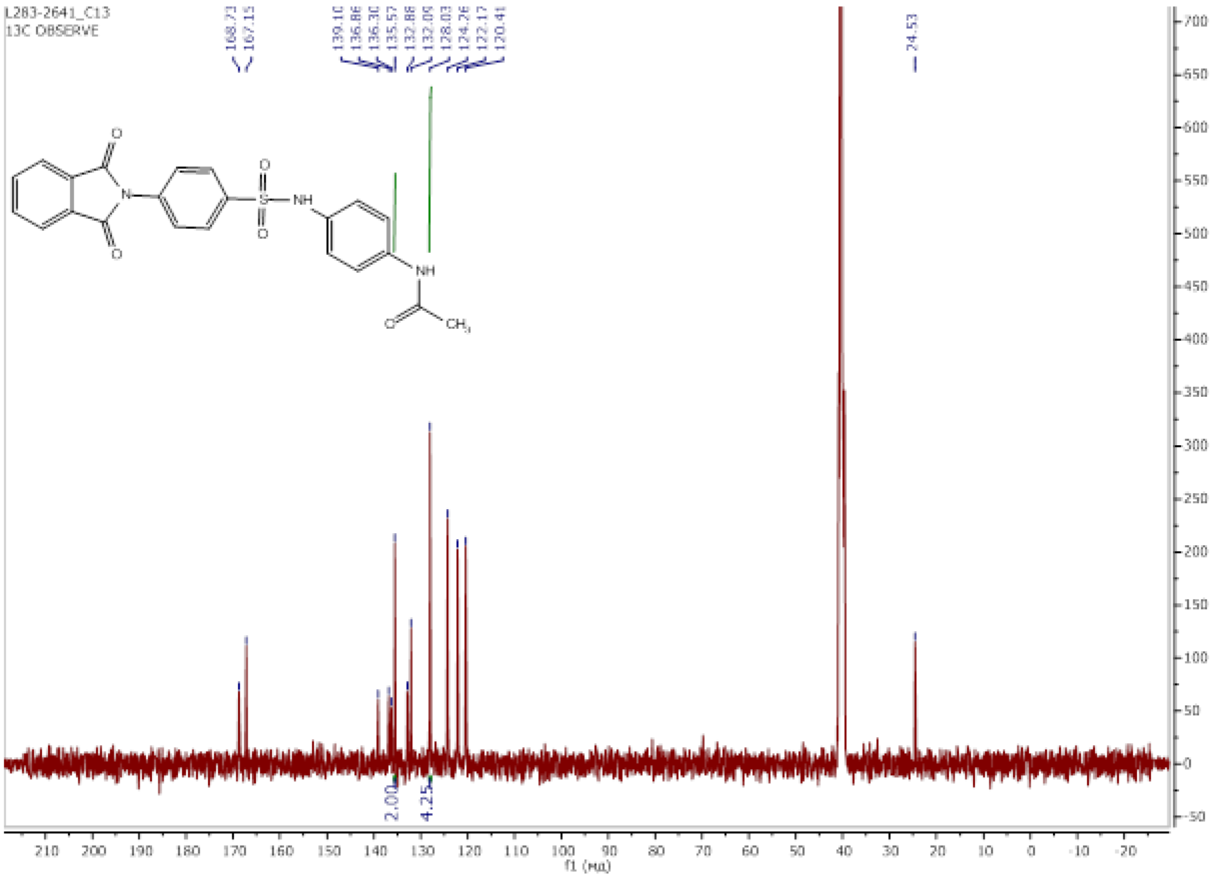

^{1}H and ^{13}C NMR spectra of 1-((4-(1,3-dioxoisoindolin-2-yl)phenyl)sulfonyl)piperidine-4-carboxylic acid **2g**

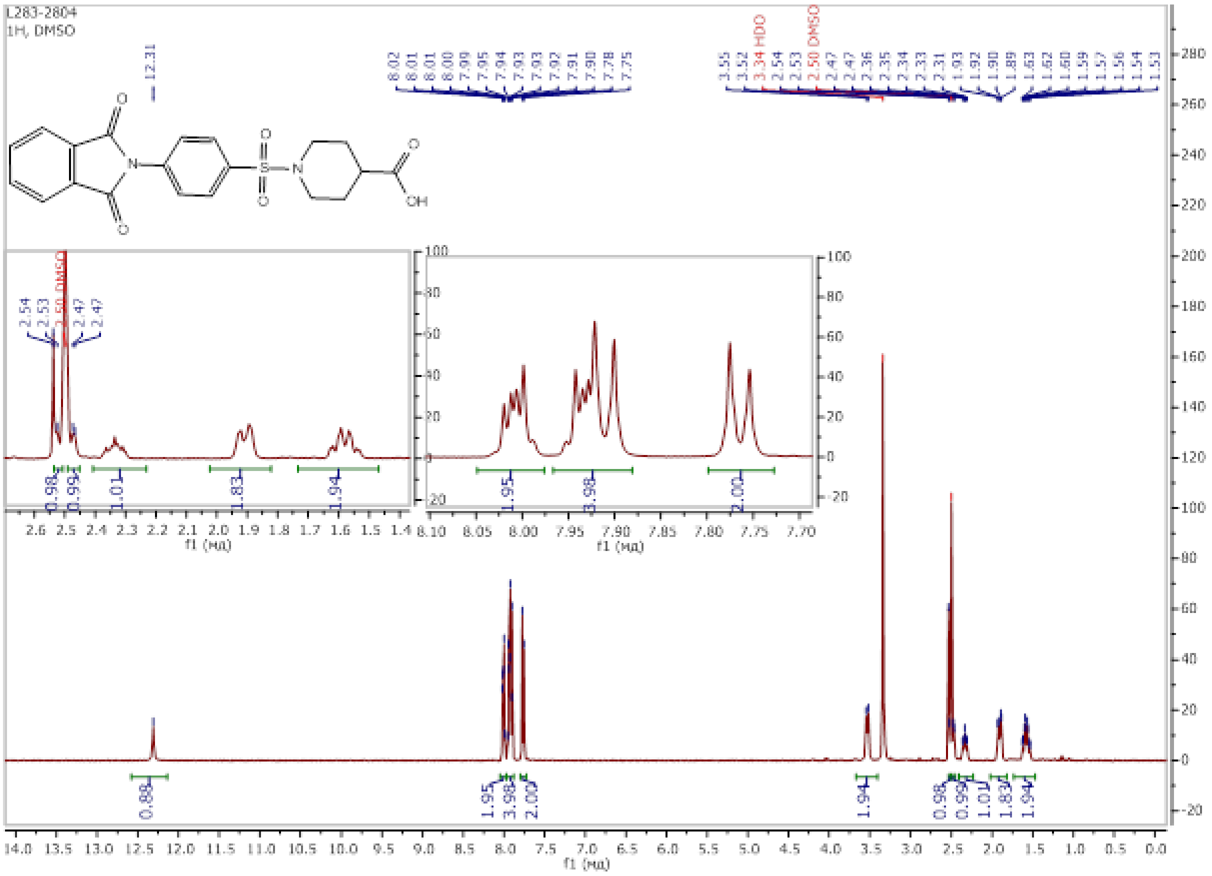

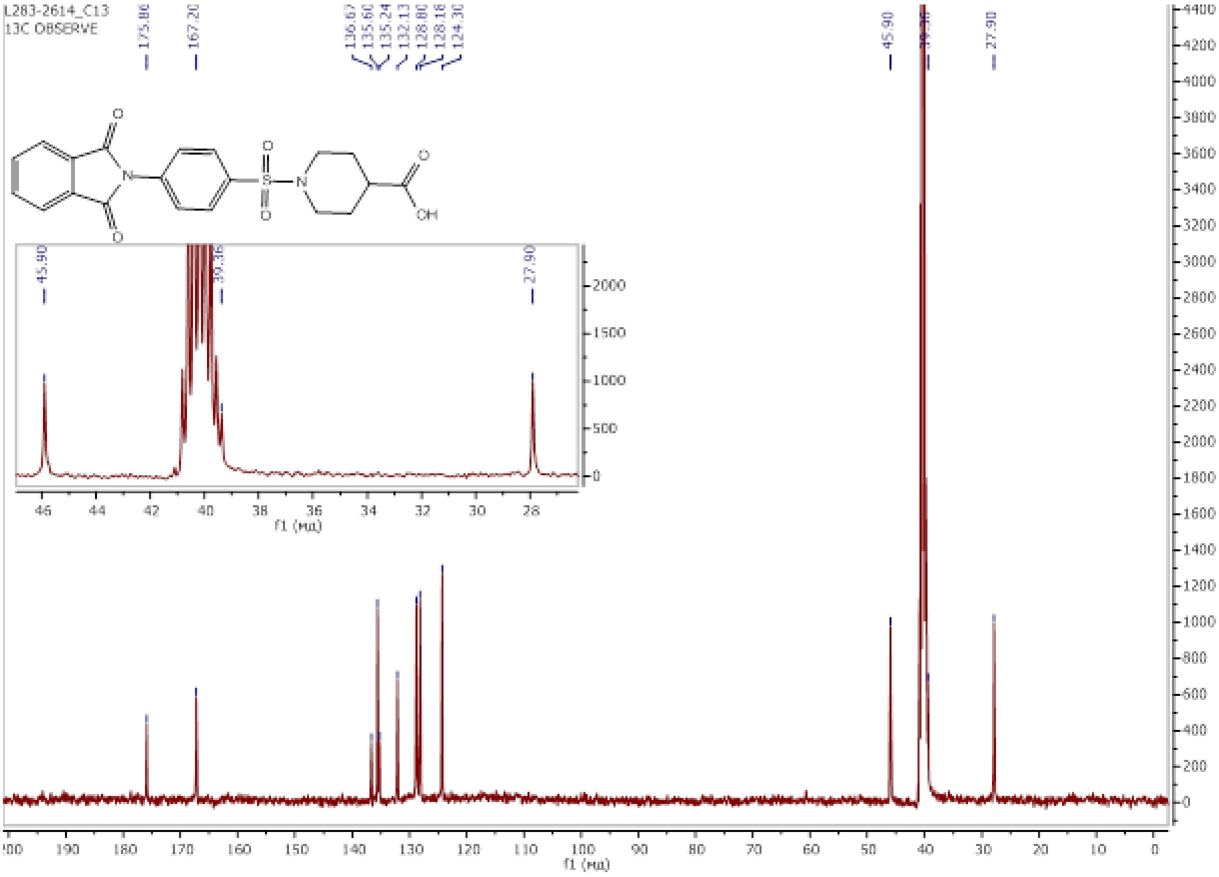

^{1}H and ^{13}C NMR spectra of 1-((4-(1,3-dioxoisoindolin-2-yl)phenyl)sulfonyl)piperidine-4-carboxamide **2j**

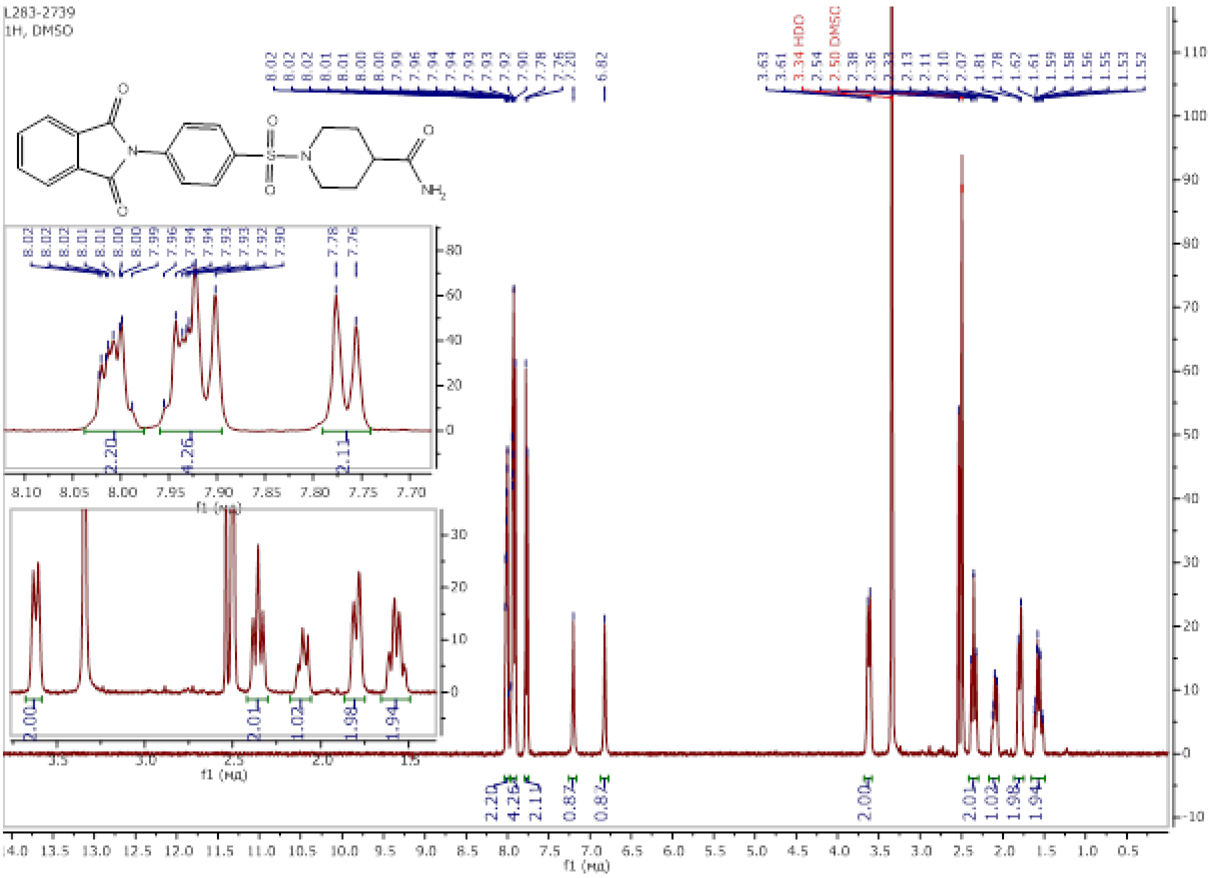

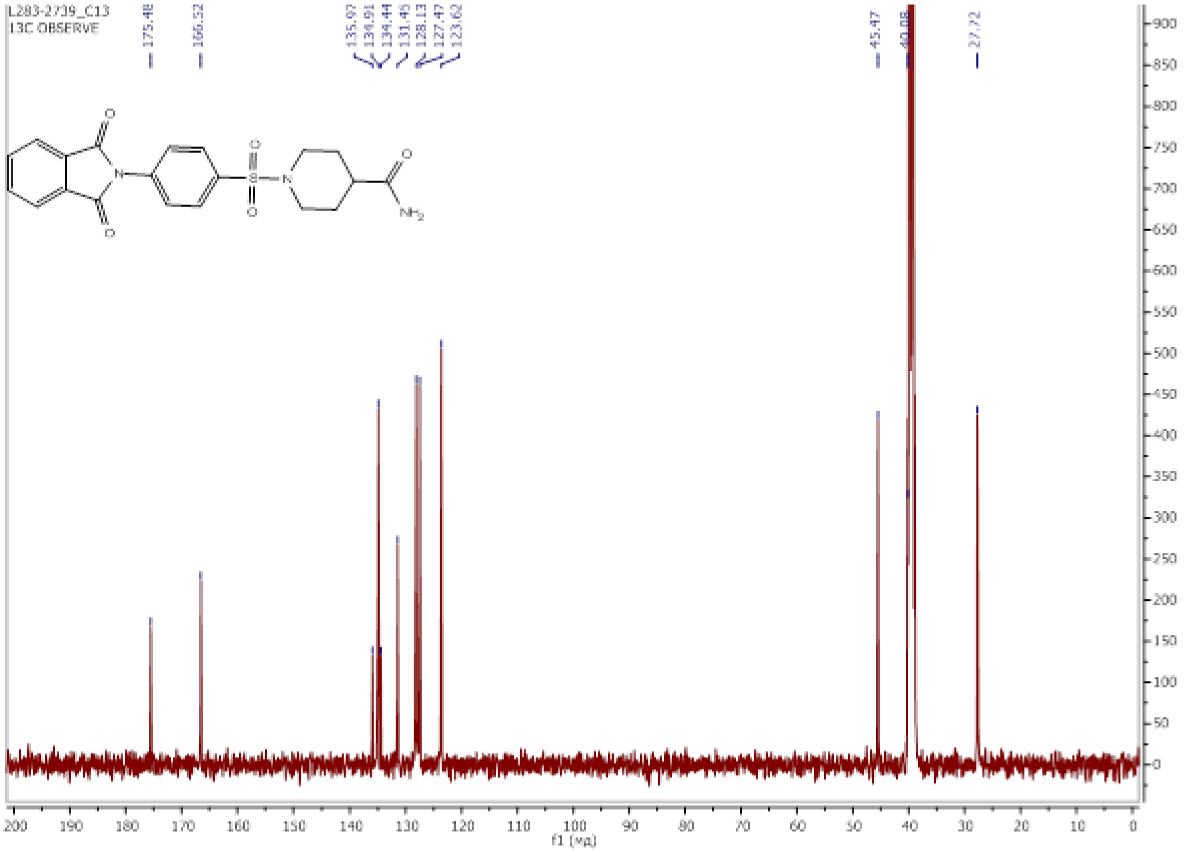

^{1}H and ^{13}C NMR spectra of 4-(4-(1,3-dioxoisoindolin-2-yl)phenylsulfonamido)butanoic acid **2i**

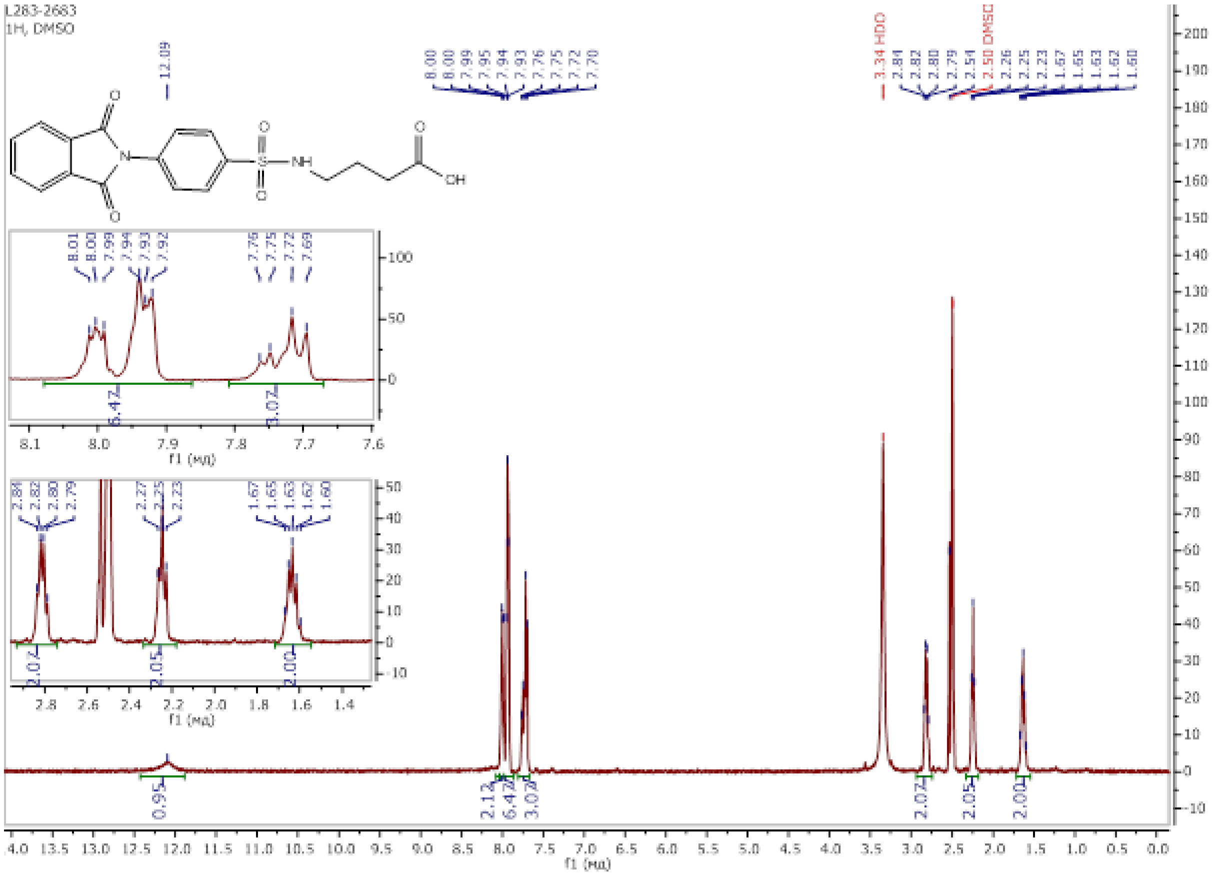

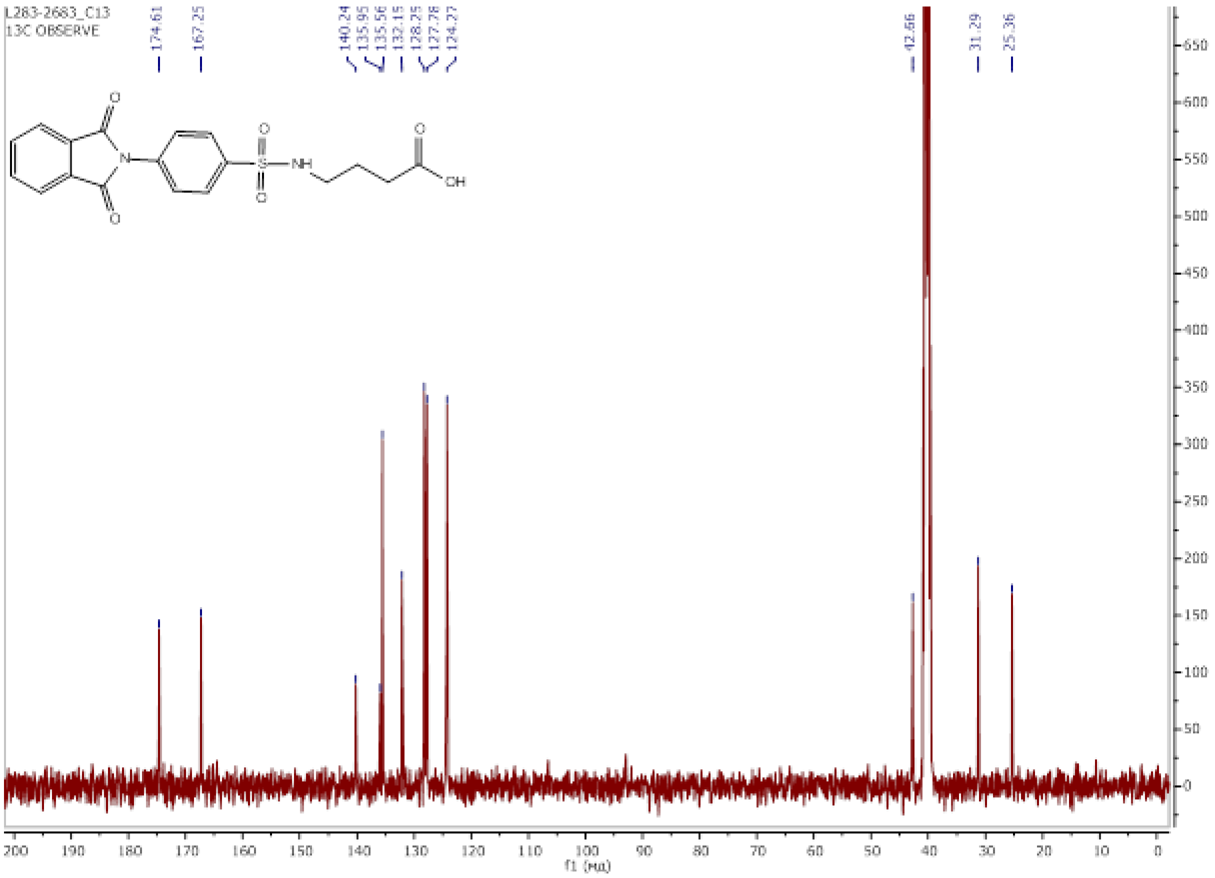

^{1}H and ^{13}C NMR spectra of 2-((4-(*N*-(4-(ethoxycarbonyl)phenyl)sulfamoyl)phenyl)carbamoyl)benzoic acid **3**

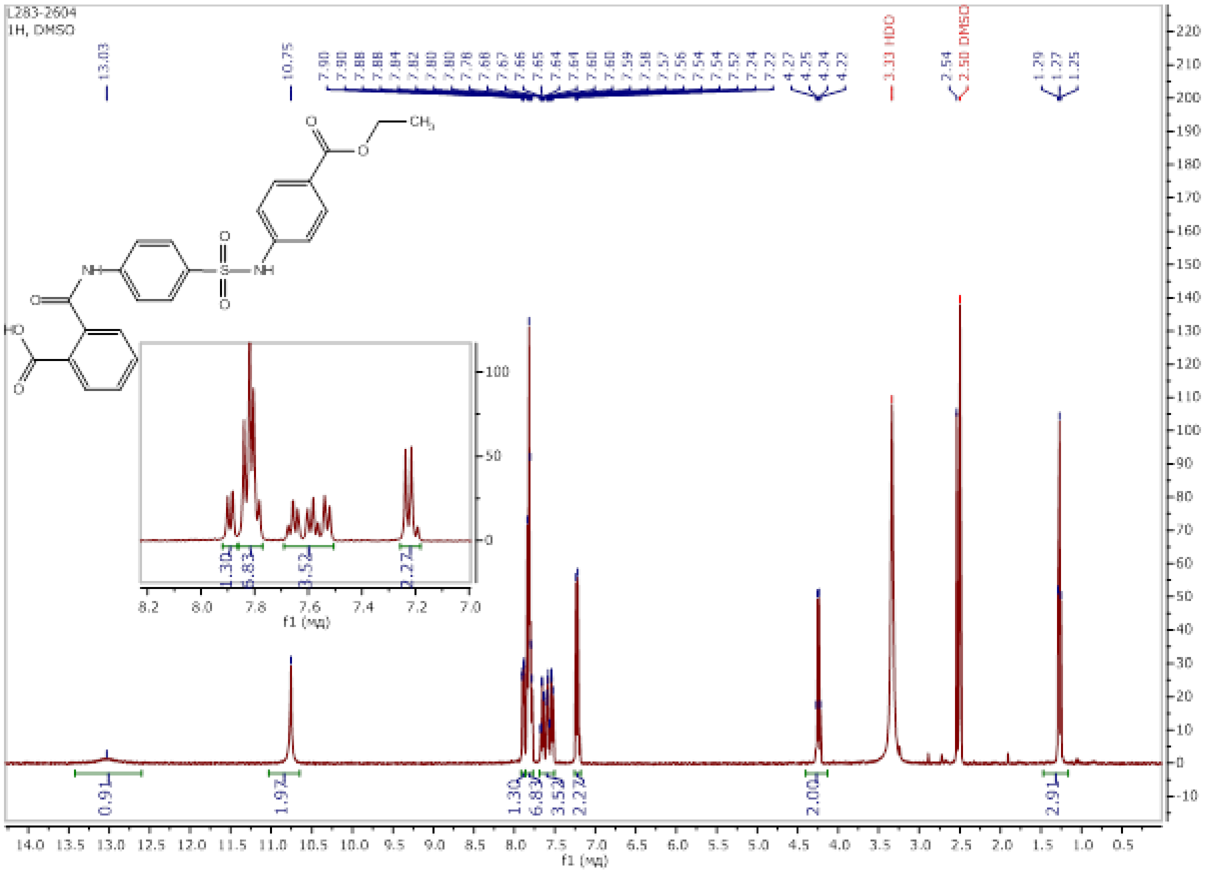

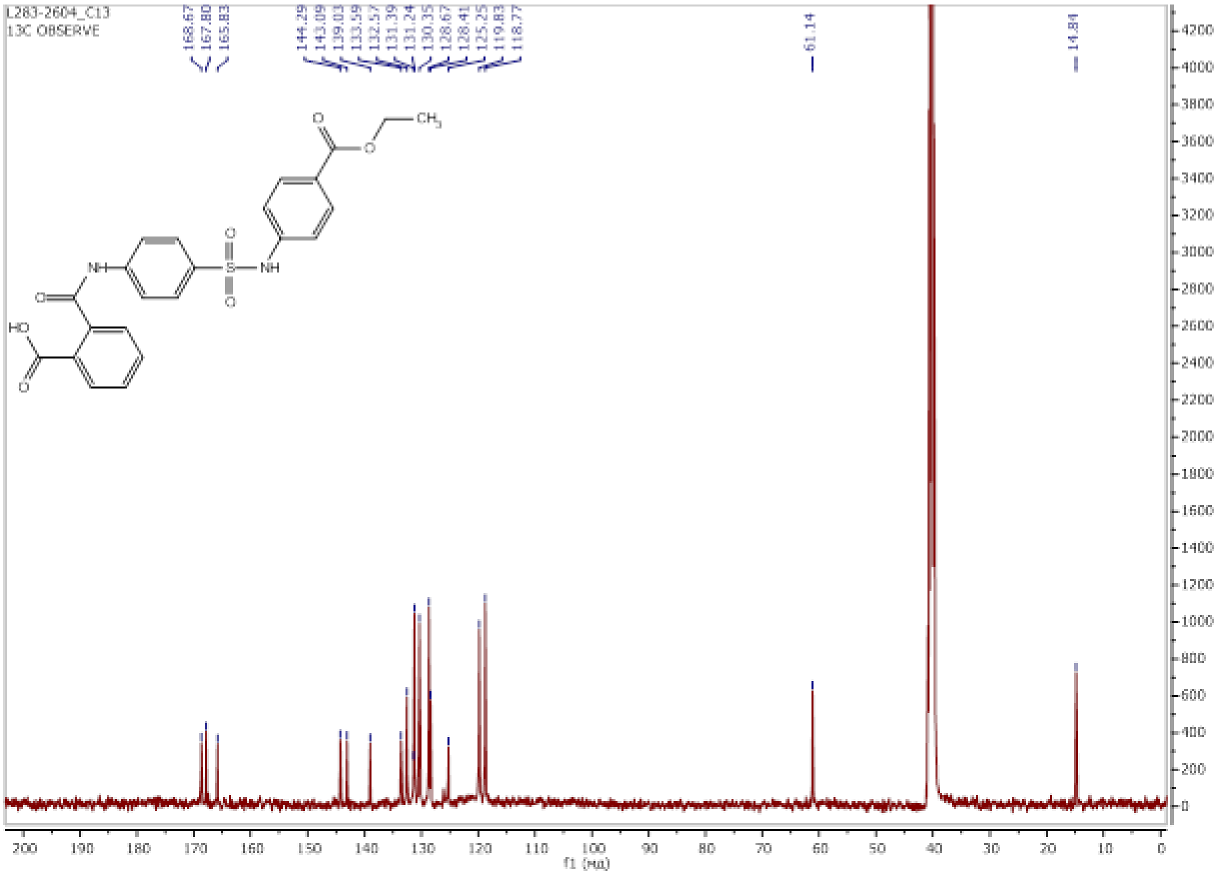

^{1}H and ^{13}C NMR spectra of 2-((4-(*N*-(4-carboxyphenyl)sulfamoyl)phenyl)carbamoyl)benzoic acid **4**

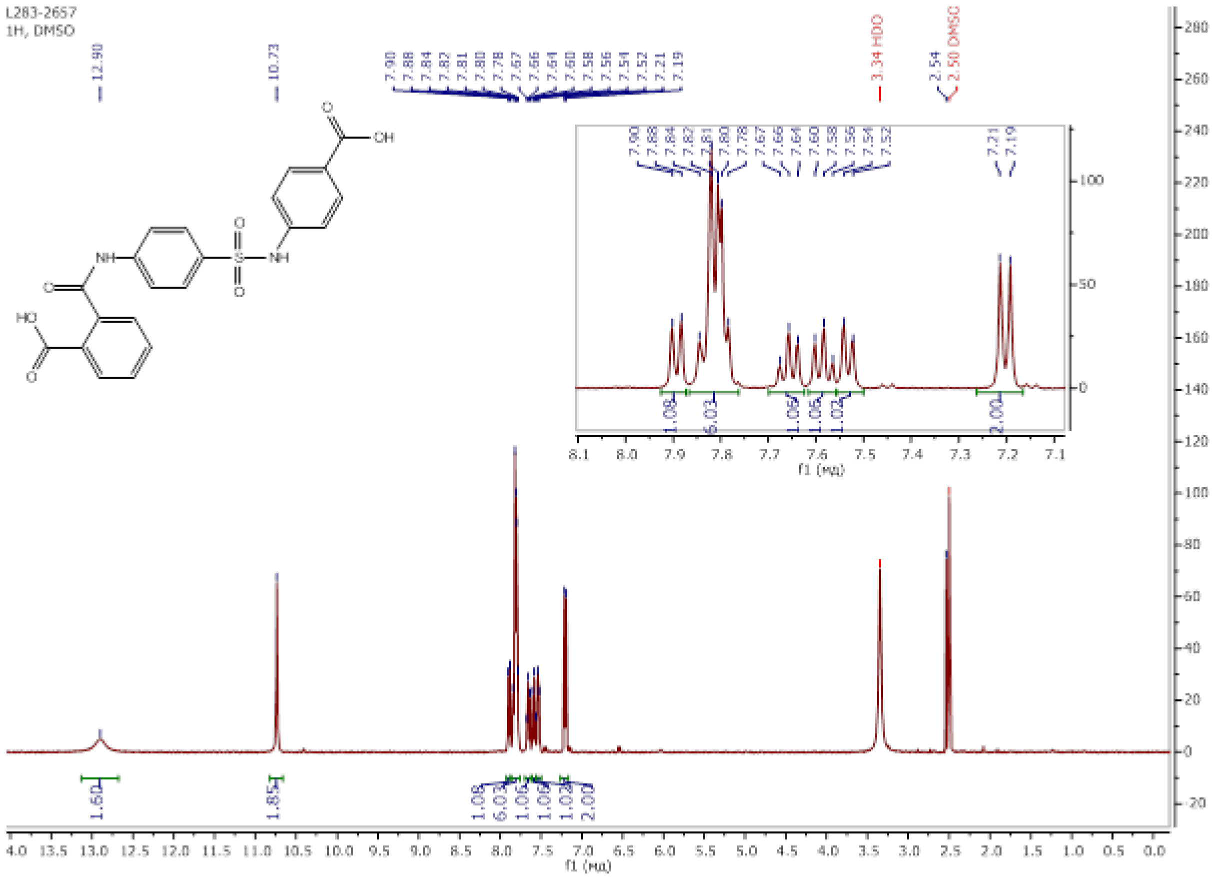

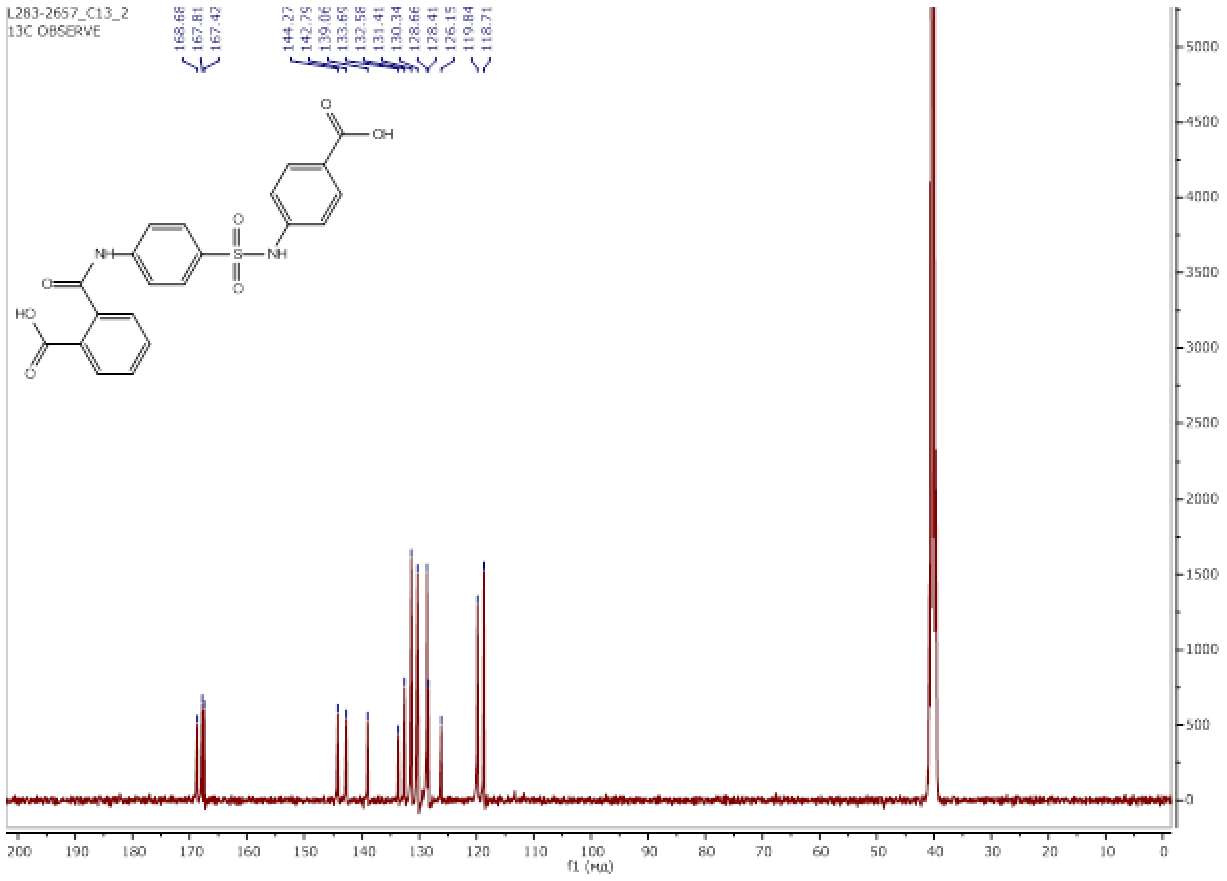

^{1}H and ^{13}C NMR spectra of 4-(4-(2,5-dioxopyrrolidin-1-yl)phenylsulfonamido)benzoic acid **7a**

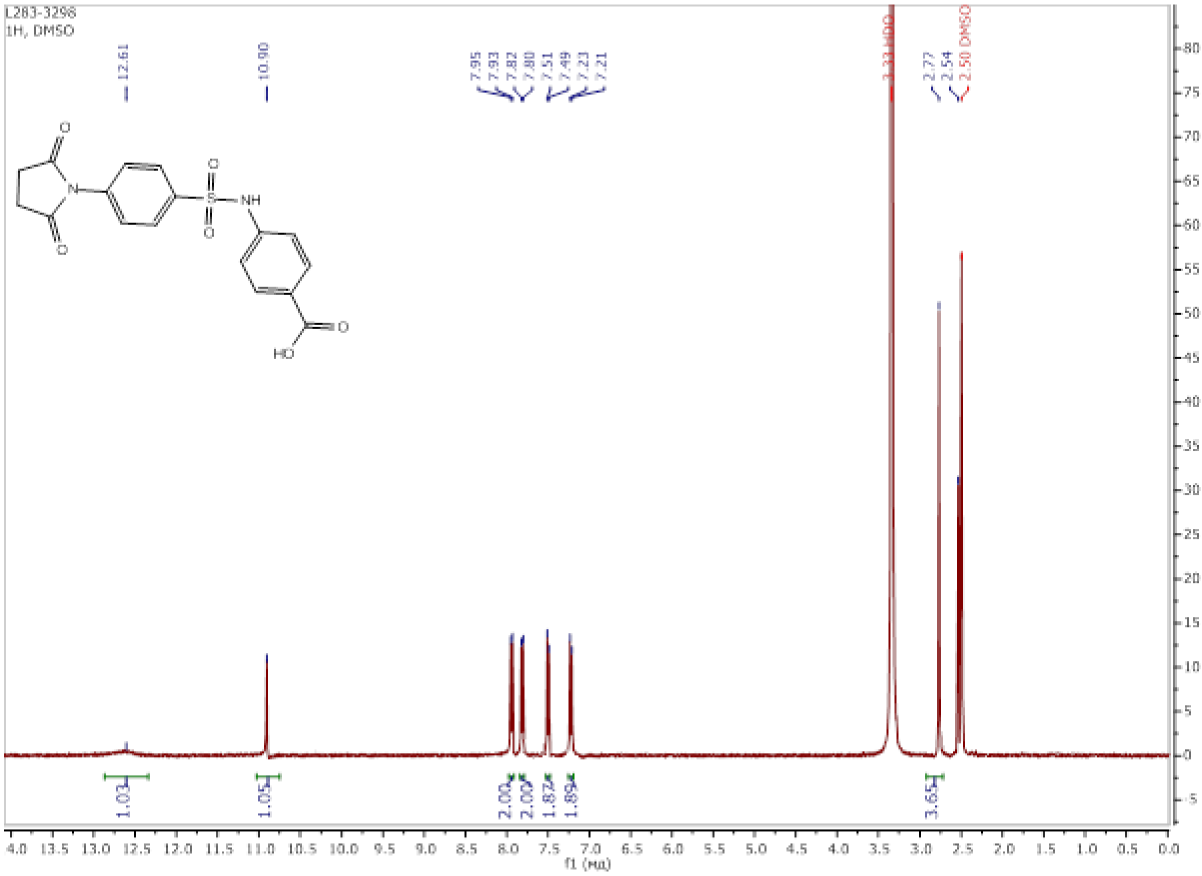

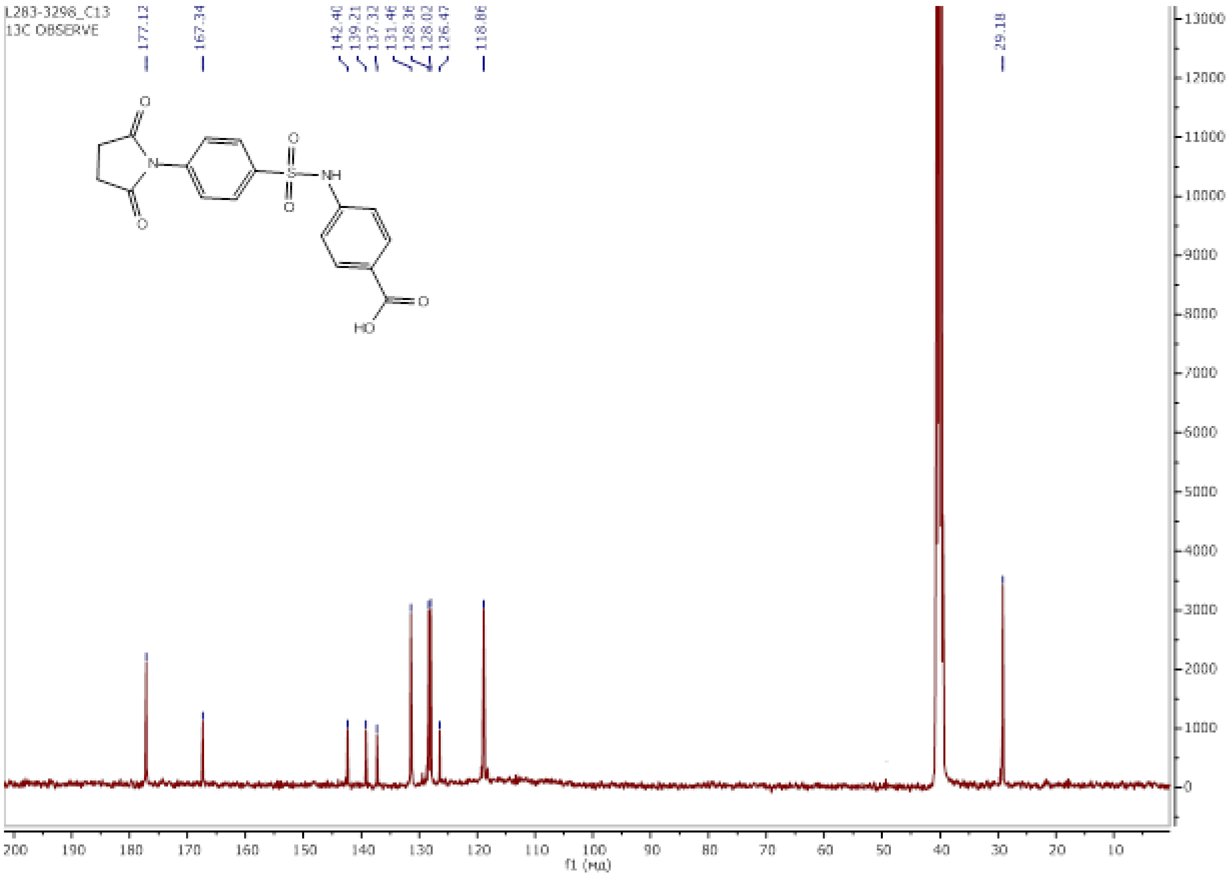

^{1}H and ^{13}C NMR spectra of 4-(4-(1,3-dioxohexahydro-1*H*-4,7-methanoisoindol-2(3*H*)-yl)phenylsulfonamido)benzoic acid **7b**

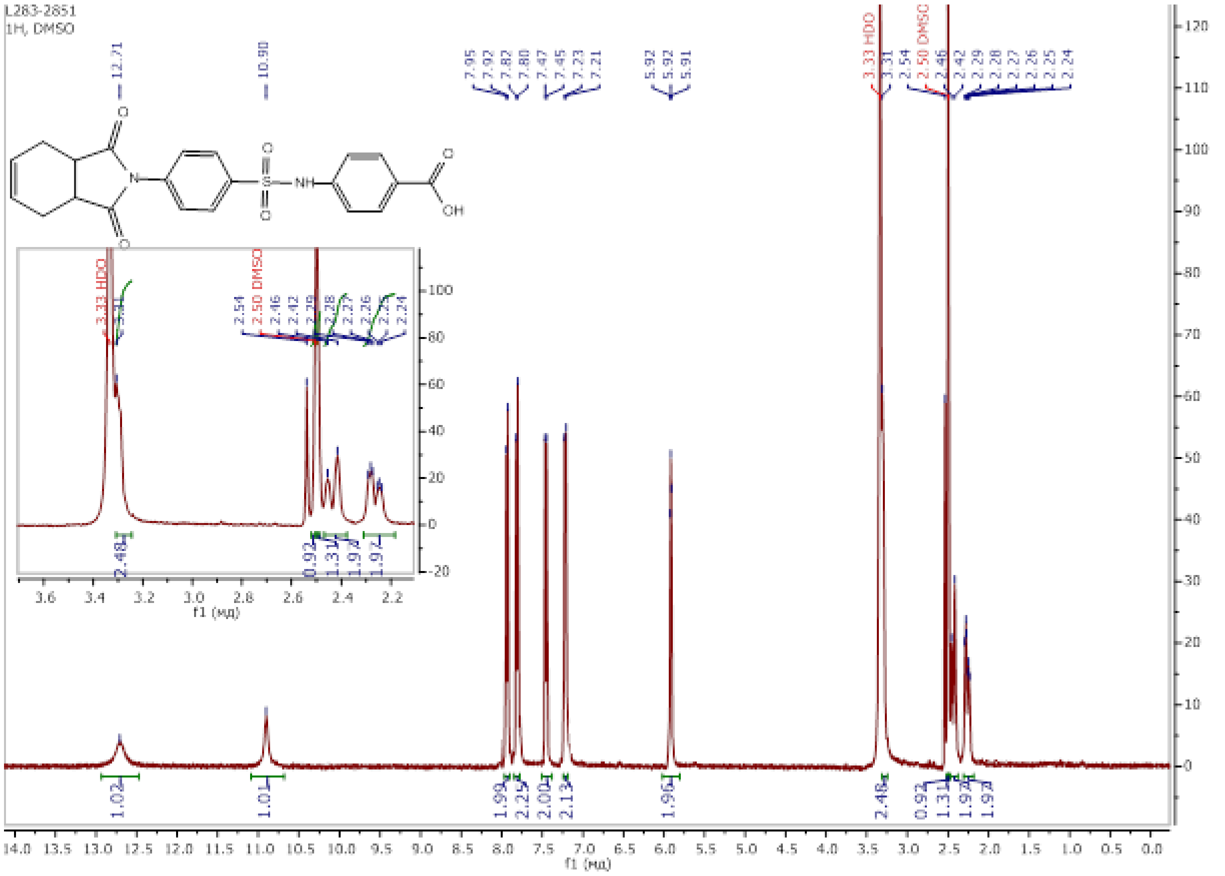

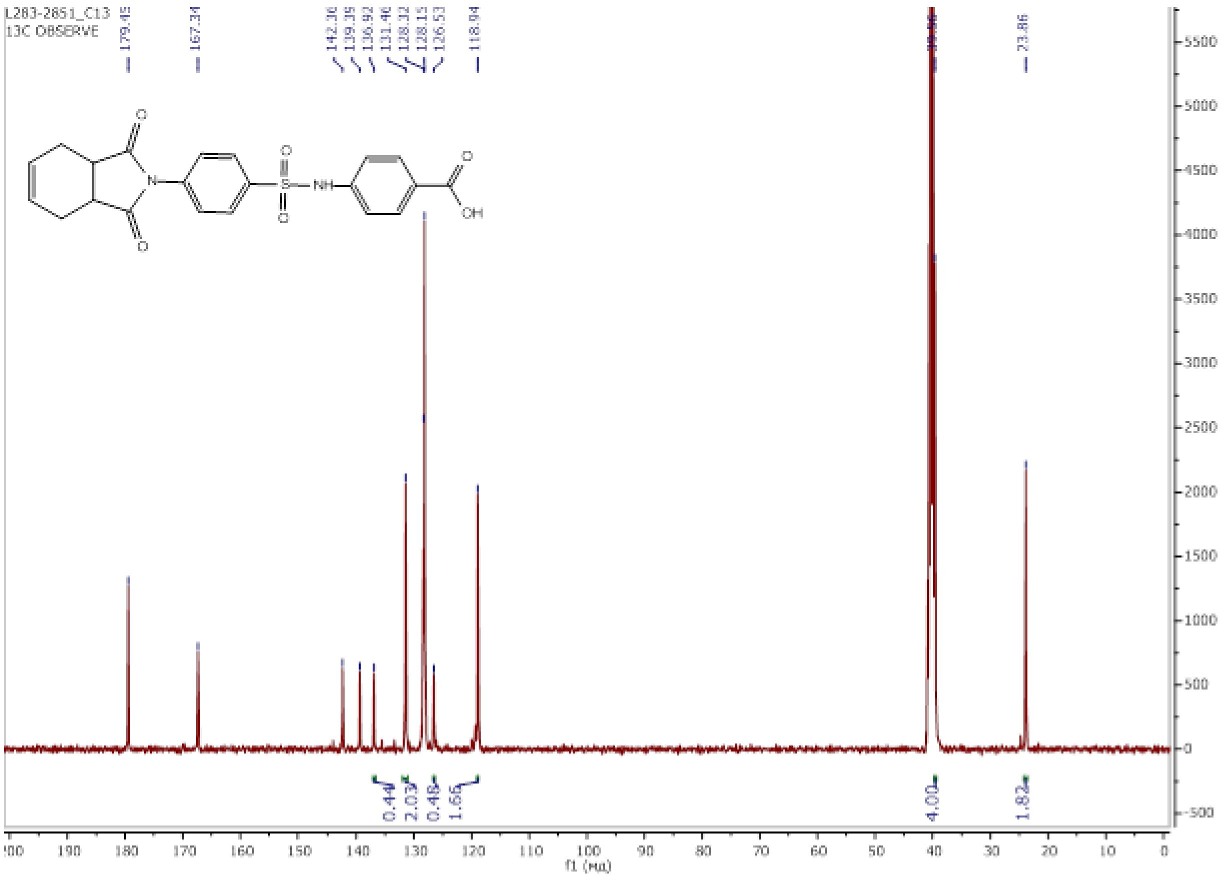

^{1}H and ^{13}C NMR spectra of 4-(4-(1,3-dioxo-3a,4,7,7a-tetrahydro-1*H*-4,7-methanoisoindol-2(3*H*)-yl)phenylsulfonamido)-benzoic acid **7c**

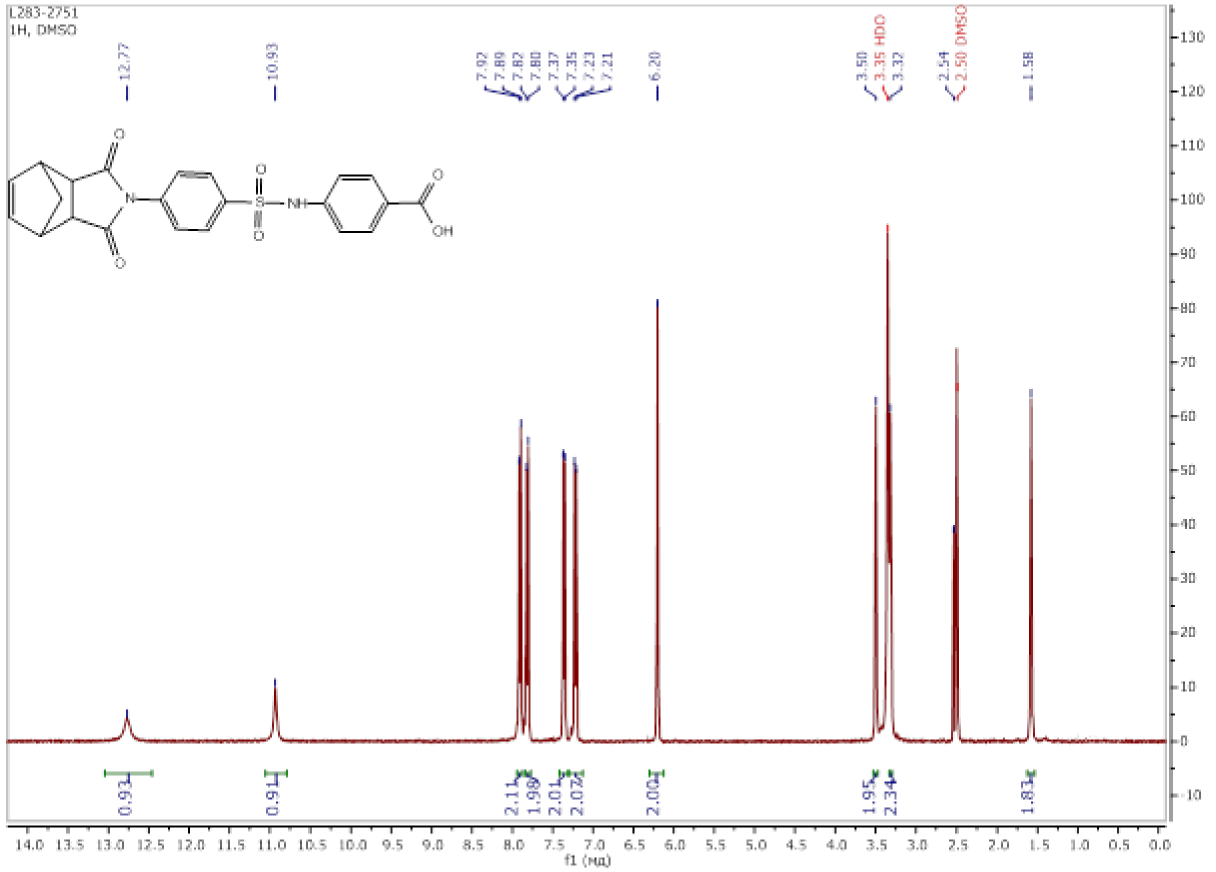

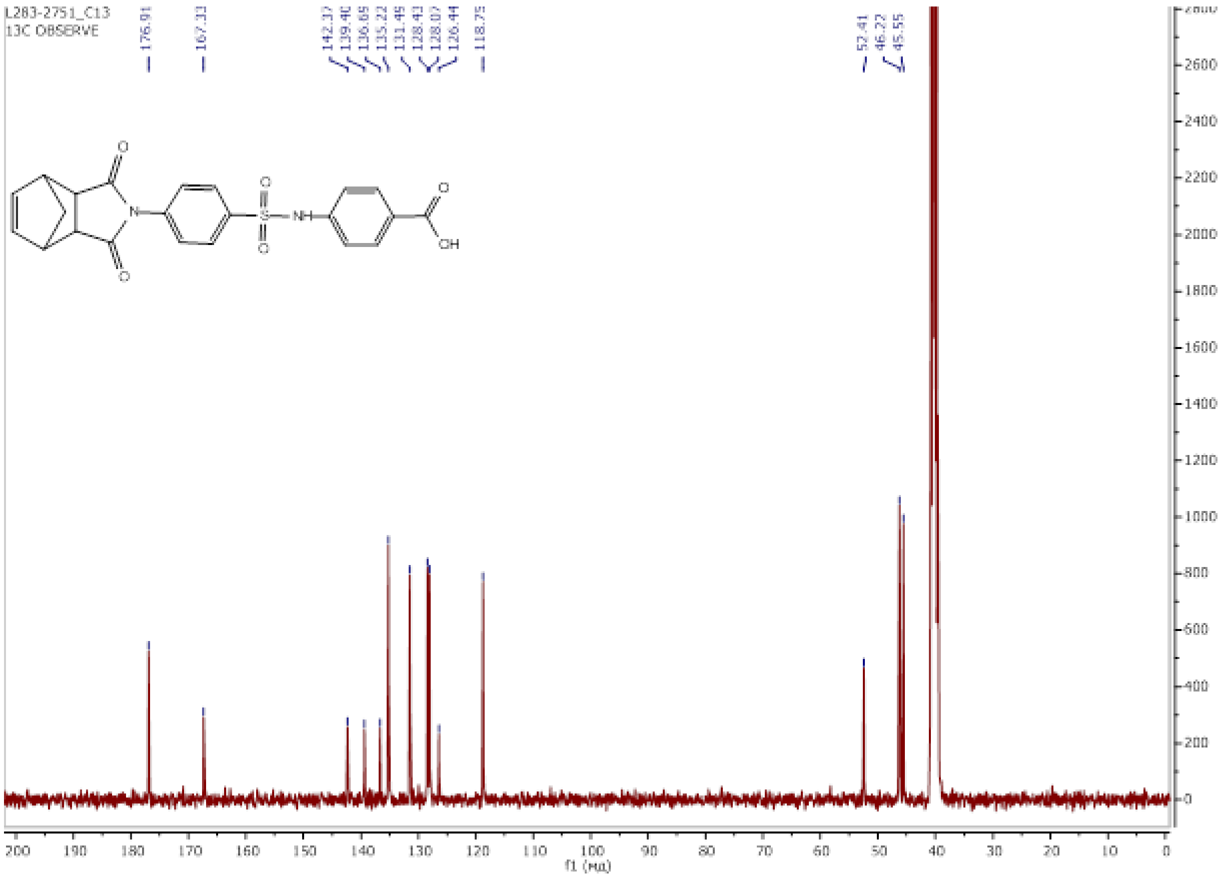

^{1}H and ^{13}C NMR spectra of 4-(4-(1,3-dioxohexahydro-1*H*-4,7-methanoisoindol-2(3*H*)-yl)phenylsulfonamido)benzoic acid **7d**

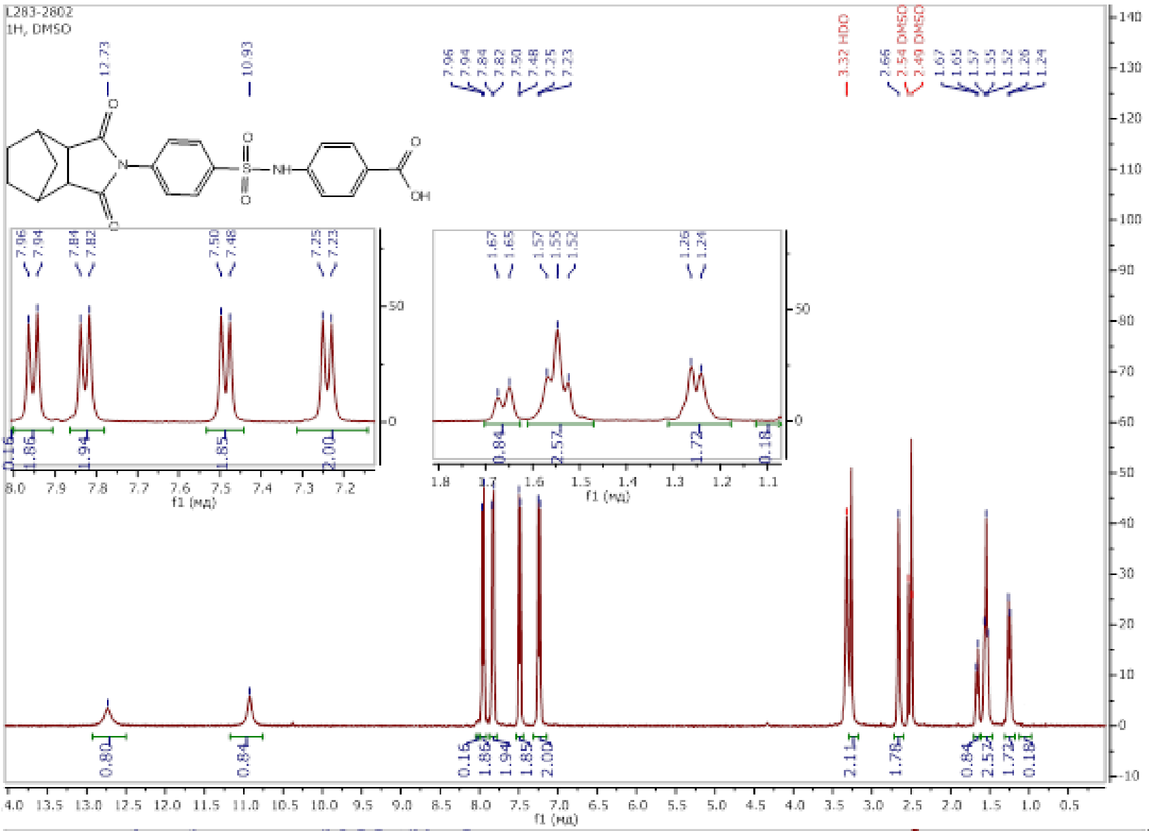

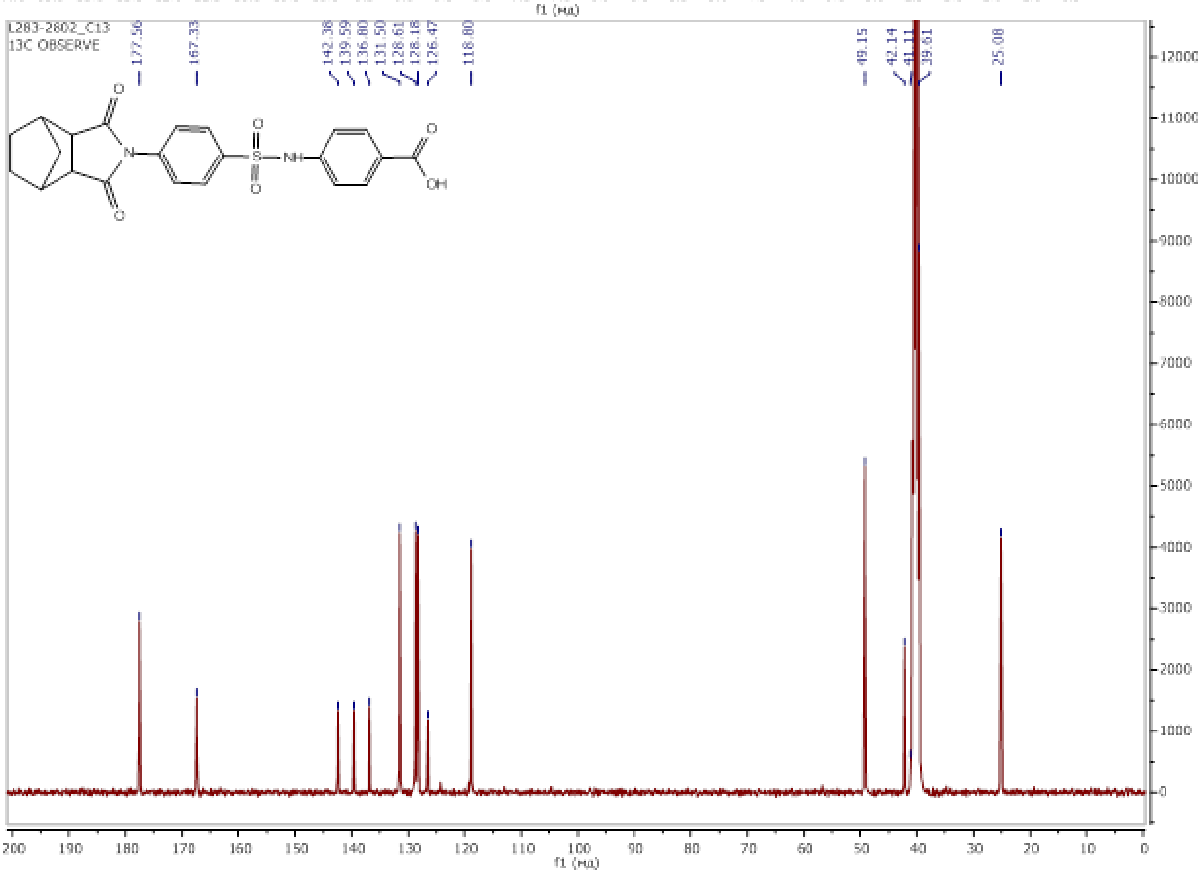

^{1}H and ^{13}C NMR spectra of 4-(4-(1,3-dioxo-1*H*-benzo[de]isoquinolin-2(3*H*)-yl)phenylsulfonamido)benzoic acid **7e**

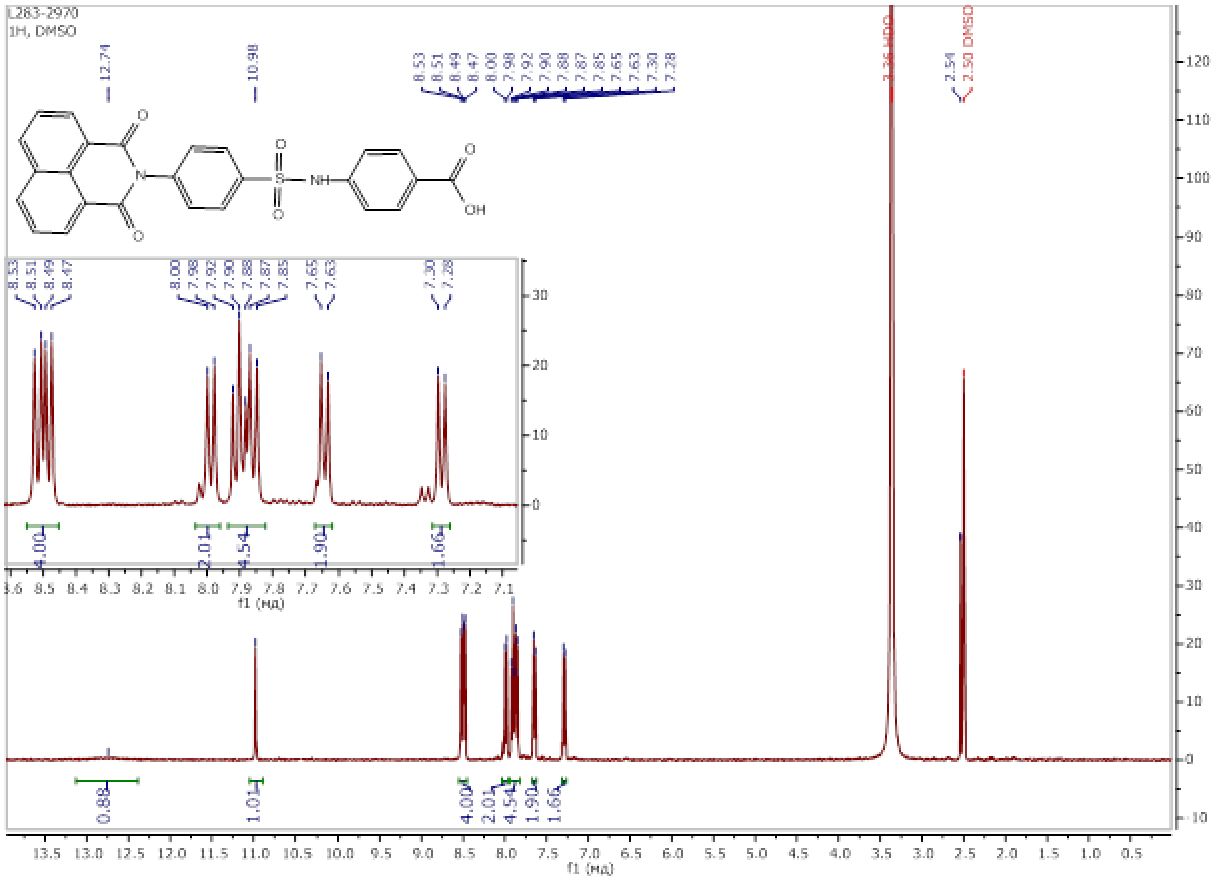

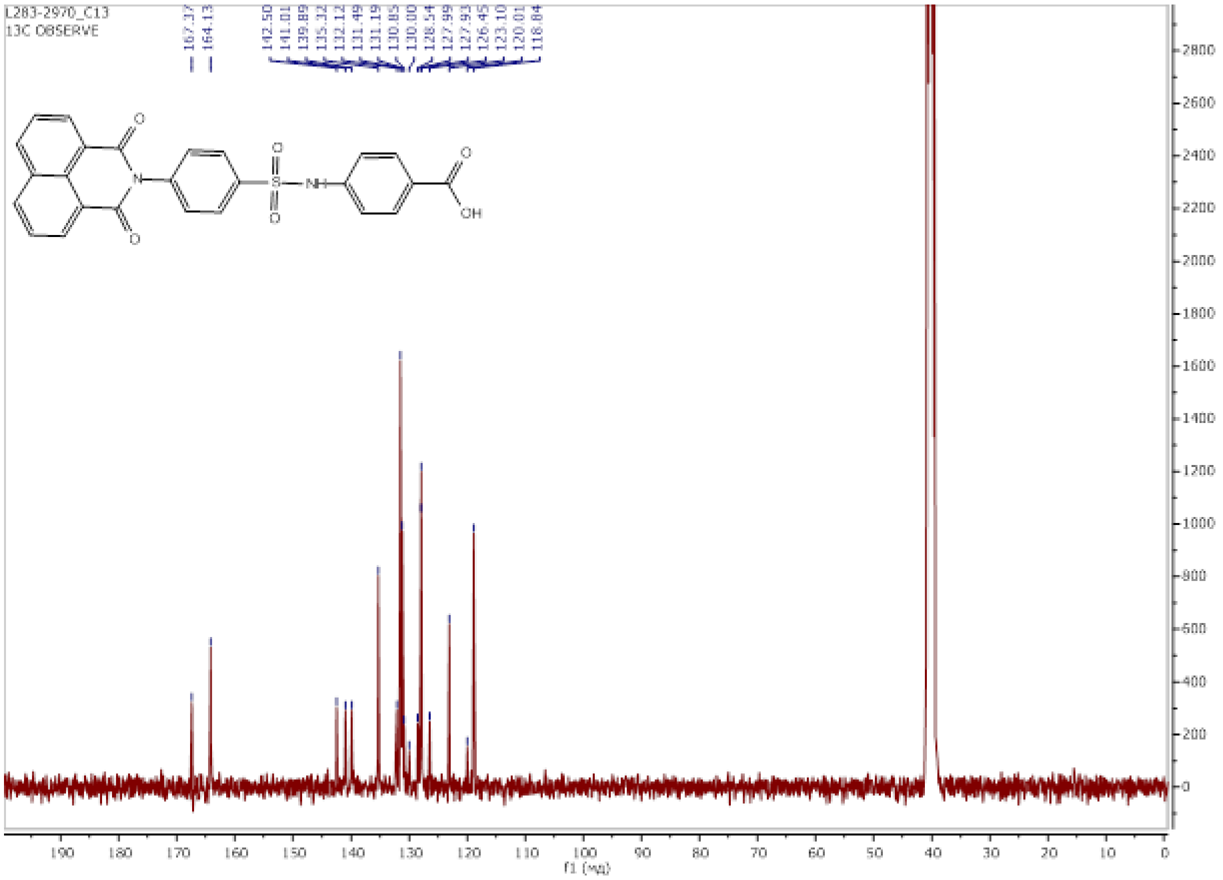

^{1}H and ^{13}C NMR spectra of 4-(4-(3-carboxypropanamido)phenylsulfonamido)benzoic acid **6a**

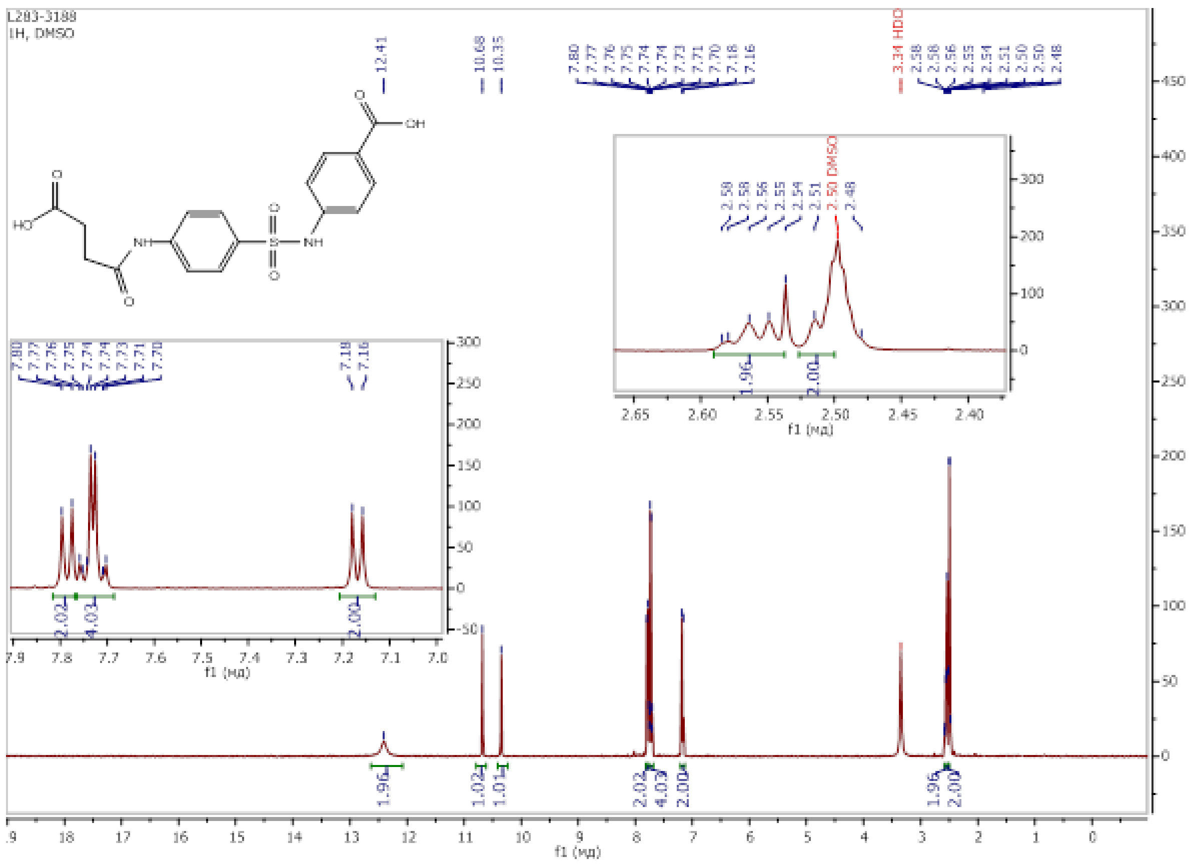

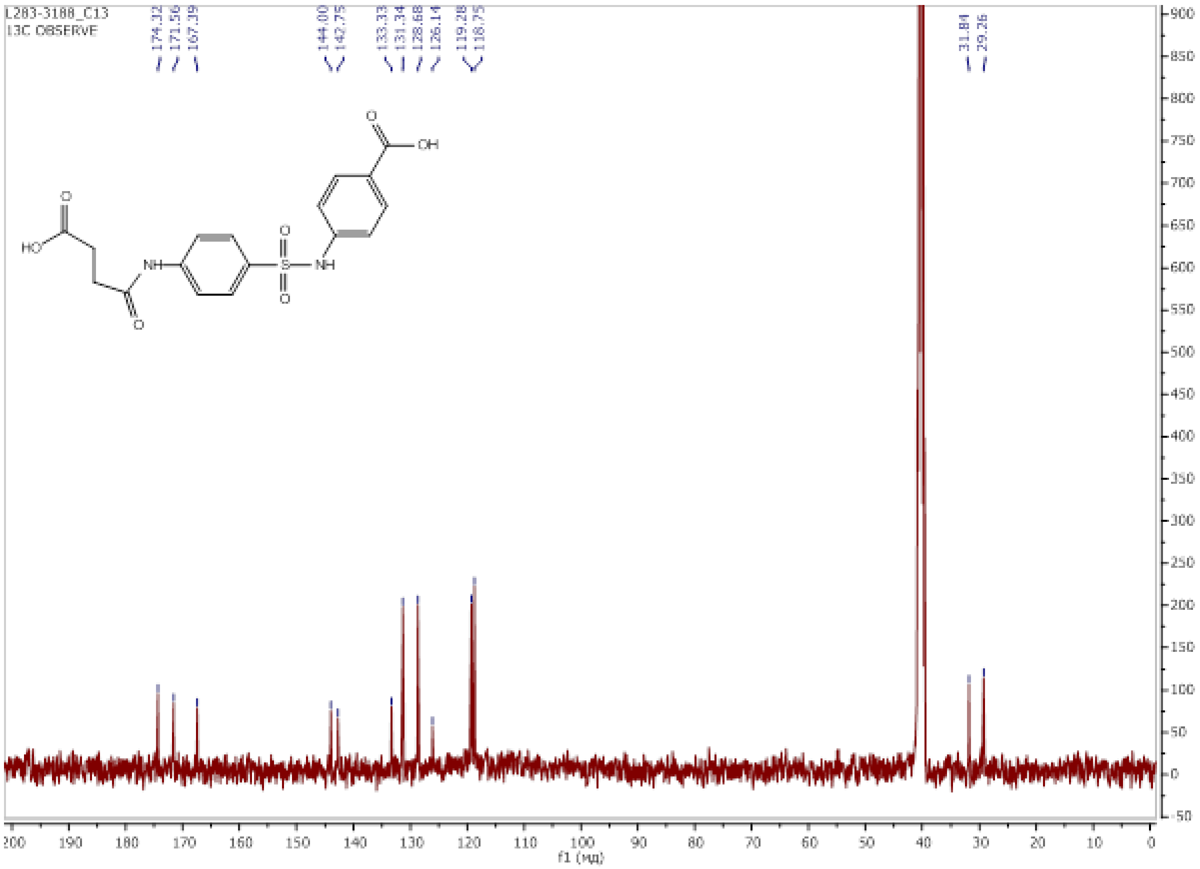

Supplement: Supplementary file 1 [file life-12-01832-s001.zip › life-2000506-supplementary.pdf]
